# Stable Isotope Dilution Analysis of the Major Prenylated Flavonoids Found in Beer, Hop Tea, and Hops

**DOI:** 10.3389/fnut.2020.619921

**Published:** 2020-12-15

**Authors:** Lance Buckett, Simone Schinko, Corinna Urmann, Herbert Riepl, Michael Rychlik

**Affiliations:** ^1^Chair of Analytical Food Chemistry, Technical University of Munich, Freising, Germany; ^2^Organic-Analytical Chemistry, Weihenstephan-Triesdorf University of Applied Sciences, Straubing, Germany; ^3^Campus Straubing for Biotechnology and Sustainability, Technical University of Munich, Straubing, Germany

**Keywords:** beer (analysis method for), stable isotope dilution analysis (SIDA), xanthohumol, xanthohumol C, isoxanthohumol, isoxanthohumol C, 8-prenylanaringeninx, 6-prenylnaringenin

## Abstract

Prenylated flavonoids from hops (*Humulus lupulus*) have become of interest in recent years due to a range of bioactivities. The potential health benefits of prenylated flavonoids include anti-cancerous activities and treatment of the metabolic syndrome among others. Since prenylated flavonoids from hops have shown pharmaceutical potential in clinical trials, robust analytical methods to determine their concentrations in food, supplements, and beverages are required. One such, the gold standard of analytical methods, is stable isotope dilution analysis due to its ability to compensate matrix effects and losses during sample work-up. As no commercial standards were available, the synthesis of seven different prenylated flavonoid isotopes utilizing various strategies (microwave assistance, acid base catalyst in the presence of deuterated substance and lastly, the use of Strykers catalyst) is described. The produced prenylated flavonoid isotopes were then applied in the first stable isotope dilution analysis method that quantified six natural prenylated flavonoids (Isoxanthohumol, Isoxanthohumol-C, 8-Prenylnaringenin, 6- Prenylnaringenin, Xanthohumol, and Xanthohumol-C) in beer, hop tea and hops to prove its applicability. The SIDA-LC-MS/MS method was validated resulting in LODs and LOQs for all analytes between 0.04 and 3.2 μg/L. Moreover, due to the simple clean-up the developed method allows the prospect for measuring clinical samples in the future.

## Introduction

**Figure 1:**
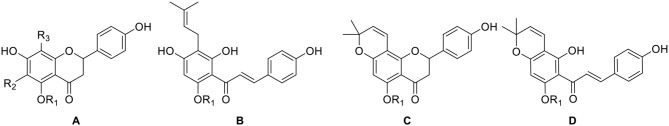
Structures of the prenylated flavonoids **(A)** Isoxanthohumol (R1=CH3, R2=H, R3=Prenyl), 8-Prenylnaringenin (R1=H, R2=H, R3=prenyl): 6-prenylnaringenin (R1=H, R2=Prenyl, R3=H). **(B)** Xanthohumol (R1=CH3). **(C)** Isoxanthohumol-C (R1=CH3). **(D)** Xanthohumol-C (R1=CH3).

Prenylated flavonoids (PF) derived from hops have undergone an increased interest in recent years regarding their potential for improving health and uses in treating various disorders. Xanthohumol (XN), the most prevalent PF in hops shows an effect on metabolic syndrome (perhaps via modulation of glucagon -like-peptide-1), prevention of DNA damage, and has anti-cancer properties (1–4) Interestingly, many of XN plant metabolites and degradation products (Figure 1) possess bioactivities (4, 5). 8-Prenylnaringenin (8-PN) is the most potent phytoestrogen known so far (6). In addition 6-Prenylnaringenin (6-PN), the isomer of 8-PN, is slightly estrogenic, but also shows an anti-proliferative effect on different cancer cell lines (7). Another PF, Xanthohumol-C (XN-C), initiates differentiation in neuronal precursor cells and is neuroprotective (8). Accordingly, it seems to be a candidate for treating neurodegenerative diseases (9). The isomerized XN-C, isoxanthohumol-C (IXN-C) shows only a slight differentiation inducing effect and accordingly, that makes the chalcone structure all the more interesting (10). Clinical trials show that PFs have a pharmaceutical use as non-modified forms (flavones) or by slight modifications e.g., removal of the Michael system (i.e., cyclization of chalcones into flavanones, or hydrogenation of the Michael system) (11–13). Further studies investigating the *in-vivo* effects are required to fully understand the beneficial properties of PF concerning different diseases and, therefore, robust and sensitive analytical methods that target the bioactive hop compounds are needed, for example, stable isotope dilution analysis (SIDA) (1). To date, SIDA has not been applied for analysis of hop PFs.

Common analytical methods applied on PF found in hops are HPLC, immunoassays, square-wave adsorptive-stripping voltammetry, high performance thin layer chromatography, MALDI, and LC-MS/MS using two different ionization sources (APCI and ESI) (1, 14–16). Some of the caveats of the current methods are: (i) they require expensive development of antibodies, (ii) are not specific enough, (iii) rely on expensive SPE clean-up, (iv) have no internal standard, or (v) use a single internal standard which might have unseen errors due to matrix effects in MS measurements (1, 17). All the disadvantages of the methods above are overcome by SIDA, the current analytical gold standard in mass spectrometry (18). The advantages of SIDA compared to LC-MS/MS using an internal standard are that it can compensate the matrix effect and work up errors as only the ratio of the isotopolog is measured. The isotope labeling of the IS, guarantees accuracy also in sample workup due to having nearly the same physical and chemical properties as the analyte i.e., losses during workup are retained as the ratio of A/IS, which is commonly accepted to be constant (18). Combined with a simple clean up, SIDA is a rapid analysis method, which is suitable for large scale intervention trials. Therefore, we aim to develop the first SIDA method on hop PFs and test the appropriateness by quantifying PF content in beer, hops, and hop tea.

As the name suggests, SIDA requires the use of isotope-labeled substances. So far, XN was isotope-labeled via multi-step synthesis or plant feeding experiments. Initially, Berwanger et al. produced a ^13^C-XN by feeding hop flower cuttings with different labeled precursors and concluded that universally labeled ^13^C glucose was the best supplement with 9.41% of the product labeled (19). Additionally, the same research group produced ^14^C XN at 318 microCi/mmol (20). More recently, (21) carried out the successful total synthesis of XN enriched with ^13^C (21, 22). The total synthesis of XN used seven steps producing a yield of 5.7%. Both of the above options are time consuming and expensive with low yields and no methods for other PFs other than XN are described. Furthermore, plant feeding experiments are generally poorly suited for SIDA, due to the presence of unlabeled isotopologues. In this study an approach to produce isotopically labeled PFs enriched with deuterium by utilizing various methods e.g., Strykers Cat, acid base catalyzation and microwave enrichment is described (23–27). The produced stable isotopes were then applied and validated using an LC-MS/MS-SIDA method to quantify the natural occurring PFs in a few selected samples of beer, hops, and hop tea.

## Materials and Methods

### General Experimental Information

All reactions were carried under inert atmosphere of argon or nitrogen unless stated. All chemicals were of technical grade unless stated. TLC monitoring was carried out during all reactions. The TLC plates used for development were silica on alumina 60 F254 (Merck). For TLC development a solvent system of 60:40 EtOAc:Hexane was utilized (Merck, Deutschland) and the stain was prepared using CAM-reagent: Ammonium molybdate tetrahydrate (Sigma aldrich) (3 g), cerium ammonium sulfate dihydrate (Sigma aldrich) (1 g), sulfuric acid 10% 100 mL (Sigma aldrich), stirred vigorously and filtered twice through filter paper (H 1/2 filter paper Schleicher & Schuell). TLC plates were dipped in the stain and heated using a heat gun until the TLC plates turned blue and dark spots formed. The analytical standards that underwent qNMR were dissolved in 600 μL MeOD-d4 (≥99.8% D, Sigma Aldrich, USA) with NMR tubes (177.8 × 4.97 mm, Bruker, USA). Staff members at the Chair of Molecular Sensory Science at the Technical University Munich carried out the NMR and qNMR experiments with a Bruker Advance II 400 MHz and 500 MHz NMR spectrometer. Data evaluation was conducted on MestReNova (Mestrelab Research, Spain) software. All samples that underwent LC-MS/MS analysis were filtered using a 0.22 μm filter (Chromafil® Xtra PVDF-20/13, 0.20 μm, Ø 13 mm; Macherey-Nagel).

### LC-MS/MS Analysis

#### Preparation of Standard Solutions

Each analytical and internal standard underwent quantitative NMR (qNMR) in methanol-d6 to determine the concentration before being made up to a final concentration of 1 mg/mL and placed in −80°C freezer. An exception is D3-8-PN and D3-XN-C the concentrations of which were calculated using HPLC-UV from calibration graphs produced by the standard compounds 8-PN and XN-C as the amounts produced in the reactions were too low for qNMR.

### LC-MS/MS Parameters

The chromatographic separation of the analytes was performed on a Shimadzu Nexera X2 UHPLC system (Shimadzu, Kyoto, Japan). The LC-parameters used solvent (A) H2O (0.1% FA) with ACN (0.1%) FA, solvent (B) with the initial solvent settings started at 25% B and increased to 35% B at 4 min where it remained until being linearly increased from 35 to 95% at 12.5 min. At 12.5 min the solvent remained at 95% B for 3.5 min until returning over 1 min back to 25%. Overall, each run took 22.5 min. The MS instrument operated in the positive electrospray ionization (ESI) mode for all analytes. The column (Restek Raptor FluoroPhenyl 2.7 μm 100 x 2.1 mm) protected with a precolumn with penta fluoro propyl phenol (PFPP) Shimadzu, Velox EXP Guard 2.1 x 5 mm) was equilibrated for 5 min between each injection. Samples were injected at 1 μL, the flow was set at 0.3 μL/min and the column oven was set to 30°C. Using an additional valve after the column, the solvent flow was introduced into the mass spectrometer at 4 min after injection to prevent the instrument from matrix contamination. The LC was coupled to a triple quadrupole mass spectrometer (LCMS-8050, Shimadzu Corporation, Kyoto, Japan). Parameters for MS were as follows: heating gas at a flow 10 L/min, nebulizing gas flow 3 L/min, drying gas flow 10 L/min, heat block temperature 400°C, desolvation line temperature 250°C, interface temperature 300°C, interface voltage 4 kV, and collision-induced dissociation gas pressure 270 kPa. The mass spectrometer worked in the scheduled multiple reaction monitoring (MRM) mode for MS/MS measurements. Optimized voltages and collision energies, the retention time of each analyte are listed in Table 1. The LabSolutions software (Shimadzu, Kyoto, Japan) was utilized for data acquisition and data analysis.

**Table 1:** The MRM profiles of the isotopologues compared with the MRM profiles of the reference compounds being analyzed.

**Analyte-D_**3**_**	**D_**3**_-XN**	**D_**3**_-IXN**	**D_**3**_-6-PN**	**D_**3**_-8-PN**	**D_**3**_-IXN-C**	**D_**3**_-XN-C**
MRM-1	358.20–180.15 (CE:−21.0)	358.25–180.05 (CE:−26.0)	345.20–289.20 (CE:−16.0)	345.20–166.15 (CE:−25.0)	356.20–235.20 (CE:−22.0)	356.20–234.20 (CE:−20.0)
MRM-2	358.20–302.20 (CE:−12.0)	358.25–302.15 (CE:−17.0)	345.20–167.10 (CE:−27.0)	345.20–167.15 (CE:−24.0)	356.20–234.70 (CE:−23)	356.20–81.10 (CE:−16.0)
MRM-3	358.20–181.15 (CE:−23.0)	358.–301.20 (CE:−15.0)	345.20–166.15 (CE:−28.0)	345.20–288.20 (CE:−16.0)	356.20–82.10 (CE:−44.0)	356.20–111.15 (CE:−33)
**Analyte-Ref**	**XN**	**IXN**	**6PN**	**8-PN**	**IXN-C**	**XN-C**
MRM-1	355.10–179.25 (CE:−21.0)	355.30–179.25 (CE:−24.0)	341.35–165.20 (CE:−29.0)	341.35–165.20 (CE:−18.0)	353.00–233.20 (CE:−22.0)	353.20–233.15 (CE:−21.0)
MRM-2	355.10–299.30 (CE:−12.0)	355.30–299.30 (CE:−15.0)	341.35–285.30 (CE:−15.0)	341.35–285.25 (CE:−15.0)	353.00–191.20 (CE:−32.0)	353.20–191.10 (CE:−31.0)
MRM-3	355.10–113.25 (CE:−23.0)	355.30–172.15 (CE:−9.0)	341.35–123.10 (CE:−37.0)	341.35–123.0 (CE:−33.0)	353.00–109.20 (CE:−35.0)	353.20–109.15 (CE−33.0)
Deuterium Enrichment	m/z +3	m/z + 3	m/z +4	m/z +4	m/z +3	m/z +3

### Analytical HPLC

The analytical HPLC analysis utilized a Shimadzu HPLC-DAD (SPA-M20A) coupled with a liquid chromatograph system (LC-20 AD, Shimadzu, Kyoto, Japan). Samples were automatically injected with an autosampler (SIL-20A HAT, Shimadzu, Kyoto, Japan) and a degassing unit (DGU-20A3R) was used when solvent system was active. Analyte separation used a C18 (YMC- Pro pack, 3 μm, 12 n, 150 x 3 mm Machery-Nagel) with a precolumn (C18, 8 x 3 mm). The mobile phase consisted of 0.1% FA in HPLC grade water as solvent A and 0.1% FA in ACN as solvent B. The flow rate was set 0.3 ml/min with gradient starting at 50% B for 2.0 min, and then gradually increasing solvent B to 90% at 11.0 min. The mobile phase remained at 90% for a further 2.5 min before being returned to 50% B. The column was equilibrated for 5 min between injections of 10 μL. Chalcones were monitored at 370 nm and flavanones at 290 nm. 8-PN was used for the calibration of D3-8-PN and XN-C was used for D3-XN-C at 0.56, 1.1, 1.7, 2.2, 2.8, 3.3, 3.9, 4.4, and 5.0 mg/L in ACN (see Supplementary Material).

### Sample Preparation

#### Beer

Fifteen beers that represented a cross section of brewery products of Bavaria were purchased from local supermarkets. They were opened and degassed using sonication. A 1 mL aliquot of each beer was placed into a 4 mL vial and IS were spiked at various concentrations, deuterated isoxanthohumol (D3-IXN) at 6.99 μM, deuterated isoxanthohumol-C (D3-IXN-C), and deuterated xanthohumol (D3-XN-C) at 0.14 μM, deuterated 8-prenylnaringenin (D3-8PN) at 0.29 μM, deuterated 6-prenylnaringenin (D3-6-PN) at 0.15 μM and deuterated xanthohumol (D3-XN) at 0.14 μM. 1 mL of EtOAc was added and the vials mixed using a vortex (Vortex Genie 2, Scientific Industries). Taking the supernatant, which was repeated 3 times, the combined organic phase of the samples was dried under N2 (set at 30°C) and resolved in 1 mL ACN.

#### Hops

Using a method derived from Stevens et al., a packet of pelletized hops (Columbus, 15.6% α-acid, USA HOPS) of a high alpha acid (15.6% AA) was purchased from the local brewing shop and ground frozen using a mortar and pestle (28). 0.1 g of the powder was taken and extracted using sonication and 100 mL of EtOAc for 15 min. Samples (0.1 mL) were spiked with IS, dried and re-dissolved in 1.0 mL of ACN.

#### Hop Tea (5%)

A brand of hop tea was purchased from the local supermarket and two different preparations were investigated. One tea bag was prepared following the instruction on the packet. 100 mL of boiling (distilled) water was added to one tea bag and brewed for 5 min. Once the tea was at room temperature the IS were spiked and the solution was dried using a N2 drier and a heating plate set at 30°C. Another sample of tea was crushed using a mortar and pestle and prepared the same as the hop pellets.

### Method Validation and Statistical Analysis

The developed method and workup procedures were validated according to the procedure of Vogelgesang and Hädrich (29). All LC-MS/MS data was collected and integrated using lab solutions software (Shimadzu Europa GmbH) and data transferred into Microsoft excel where all calculations were performed using excel functions (Microsoft Corporation, USA).

### Preparation of Calibration Graphs

Calibrators of all 6 analytes (Reference standards) were spiked against their internal standards (IS) in ACN at the following molar ratios [n(Ref)/n(IS)]: IXN, 14 points: 100:1, 80:1, 60:1, 40:1, 20:1, 10:1, 7.50:1, 1:1, 1:20, 1:40, 1:60, 1:80, 1:100 (*R*^2^ = 0.9982). IXN-C 12 points: 100:1, 60:1, 25:1, 10:1, 7.5:1, 5:1,1:1, 1:20, 1:40, 1:60, 1:80, 1:100. (*R*^2^ = 1). 8-PN, 14 points: 25:1, 15.:1, 12.5:1, 9.7:1, 7.5:1, 5.0:1, 4.4:1, 3.9:1, 3.3:1, 2.8:1 1.7:1, 0.1:1, 0.05:1, 0.04:1 (*R*^2^ = 0.9995). 6-PN 11 points: 20:1, 15:1, 12.5:1, 5.9:1, 1.98:1, 1.5:1, 1.3:1 0.65:1, 0.43:1, 0.2:1, 0.002:1. (*R* = 0.9999). XN, 11 points: 100:1, 80:1, 60:1, 40:1, 20:1, 1:1, 1:20, 1:40, 1:60, 1:80, 1:100 (*R*^2^ = 0.9996). XN-C, 10 points: 80:1, 60:1, 40:1, 20:1, 1:1, 1:20, 1:40, 1:60, 1:80, 1:100 (*R*^2^ 0.9997). The calibration graphs were formed from the LC-MS/MS data collected and from integration of the area of each peak using lab solutions software (Shimadzu Europa GmbH). The area of each peak A(ref)/A(IS) was then plotted into Microsoft excel (Microsoft Corporation, USA) against the known n(ref)/n(IS). Mandel testing confirmed linearity of the graphs and the equation resulted in determining the concentration of the prenylated flavonoids found in the samples (30). See Supplementary Material for each plotted calibration graph. Each calibrant level was injected 3 times and are the average value reported.

### Precession, Limits of Detection and Limits of Quantification

The LOD concentrations were initially estimated by DIN 32645 (31). A hop free beer, a kindly gift of the TUM Research Center Weihenstephan for Brewing and Food Quality., served as blank matrix. Activated charcoal 1 g was added to the hop free beer (50 mL) and the solution was left to stirring for 1 h before being filtered through filter paper. The hop free beer was extracted three times with EtOAc (50 mL) and then concentrated to dryness and dissolved in ACN. The blank matrix was then spiked with the corresponding amounts of analytes near the estimated LOD to apply the method by Vogelgesang and Hädrich (29): IXN (0.18, 0.70, 1.3, 1.8 μg/L, and IS at 1 μg/L), IXN-C (0.05, 0.35, 0.2, 0.5 μg/L, and IS at 0.5 μg/L), 8-PN and XN (0.25, 1.0, 1.8, 2.5 μg/L, and IS at 2.0 μg/L), 6-PN (0.16, 0.64, 1.1, 1.6 μg/L, and IS at 1.0 μg/L), XN-C (0.2 μg/L, 0.8 μg/L, 1.4 μg/L, 2.0 μg/L and IS at 0.5 μg/L). For recoveries the matrix was spiked at 4 different levels (2 low and 2 high). IXN low level, 0.004, 0.009 mg/L (D_3_-IXN 0.0025 mg/L), and high 2.5, 4.0 mg/L (D_3_-IXN at 2.5 mg/L). IXN-C low level, 0.004, 0.009 mg/L (D_3_-IXN-C 0.0025 mg/L), high level 0.5, 0.05 mg/L (D_3_-IXN-C 0.1 mg/L). 8-PN, low level 0.004, 0.009 mg/L (D_3_-8-PN, 0.0025 mg/L). High level 0.5, 0.25 (D_3_-8PN, 0.1 mg/L). 6-PN low level 0.004, 0.009 mg/L (D_3_-6-PN 0.00125 mg/L). High level 0.5, 1.0 mg/L (D_3_-6PN 0.25 mg/L). XN, low level 0.004, 0.009 mg/L (D_3_-XN 0.0025), and high level 0.5, 1.0 mg/L (D_3_-XN 0.5 mg/L) XN-C low level, 0.004 mg/L and 0.009 mg/L (D_3_-XN-C: 0.0025 mg/L), and high level 0.025, 0.05 (D_3_-XN-C 0.05 mg/L). Each sample underwent the same extraction in section Beer, and molarity of each compound was calculated using linear regression using the calibrations graphs prepared above. The ratios of the theoretical spiked vs. the calculated values determined the recoveries, LOD and LOQ. Precision was checked by triplicate determination of a mixed sample being injected 9 times and the RSD of ratios of the A(ref)/A(IS) calculated. Intra-daily precision was determined by measuring 3 samples twice during one day and the inter-daily precision was determined by measuring a sample 3 times weekly using double injections.

### Calculation of the Prenylated Flavonoid Content

The concentration of each analyte in each sample was calculated by taking the integrated area ratio of the analyzed reference compound (unknown) compared to the peak area of the spiked internal standard. The ratio of A(ref)/A(IS) was then used with linear regression equation reported in each excel graph to calculate the concentration [i.e., n(unknown)/n(IS)] in the samples in triplicate +− SD. The total PF content was calculated from the sum of each analyte using the excel function. The concentration of the PF in samples are expressed as the mean +− SD.

### Analytical HPLC

To characterize and quantify labeled XN-C and 8-PN analytical HPLC was necessary. HPLC using a LC-20 AD Shimadzu Prominence HPLC System (Shidmadzu Kyoto, Japan) coupled with a SPA-M20A prominence diode array detector. And a YMC- Pack Pro C18, S-3 μm, 12 n, 150 x 3 mml. D, precolumn: 8 x 3 mm (Machery-Nagel, Düren, Germany) column. The retention times and UV profiles were compared with the non-deuterated compound (see Supplementary Material). The non-deuterated forms of XN-C and 8-PN were constructed into calibration solutions of 5.0, 4.4, 3.9, 3.3, 2.8, 2.2, 1.7, 1.1, and 0.6 mg/L. Upon evaluation of the graphs equations of XN-C y = 0.00001x + 0.0724, *R*^2^ = 0.998 and 8-PN y = 42810x + 1007, *R*^2^ = 0.999 were used to quantify the concentration of the D3-XN-C and D3 8-PN.

## Results and Discussion

### Synthetic Results

**Figure 2:**
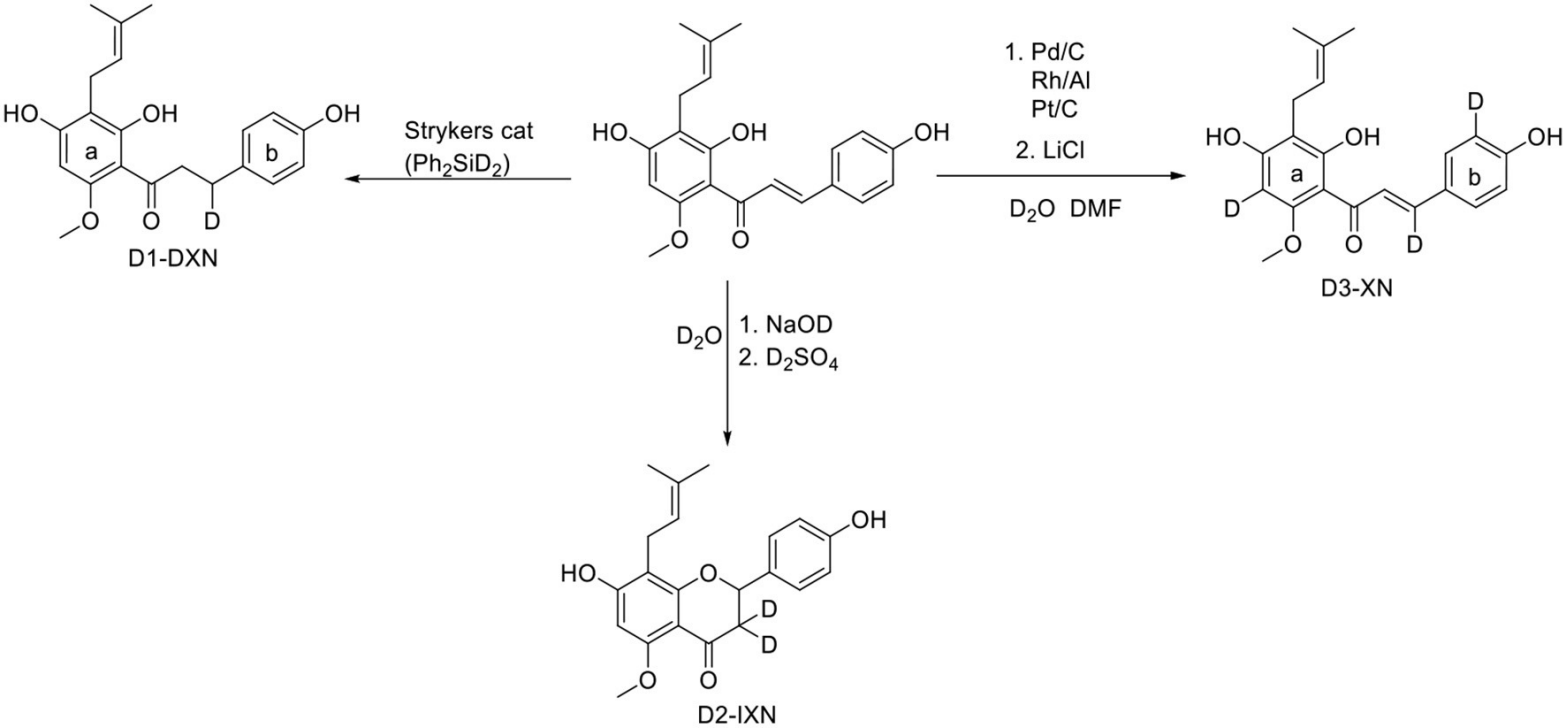
The synthetic approaches applied to synthesize isotope labeled prenylated flavonoids.

As no commercial isotopically labeled standard of any prenylated hop flavonoid were available all isotopologues were synthesized (Figure 2) before SIDA was carried out (for detailed synthesis instruction see Supplementary Material). First, the isotope labeled standard of IXN was synthesized using wet chemistry, cyclisation following (32), with all protic solvents replaced by deuterated equivalences (32). To produce isotopically labeled xanthohumol (D_3_-XN), 6-prenylnaringenin (D_3_-6PN), 8-prenylnaringenin (D_3_-8PN) and isoxanthohumol (D_3_-IXN) two synthetic methods were combined. One, the removal of aryl methyl ethers by LiCl in DMF and the other, enrichment of deuterium by D_2_O and catalyst mixtures of Pt (30%)/C, Pd (30%)/C, Rh (10%)/Al. (26, 33). Using this approach, four isotopically labeled standards (D_3_-IXN, D_3_-6-PN, D_3_, 8-PN, and D_3_-XN) were created in 1 pot (Supplementary Material) reaction. A recent method using the demethylation and microwave approach has greatly improved the yields for production of non-deuterated 6- and 8-PN (27). The synthesized D_3_-XN and deuterated IXN (by wet chemistry approach) were then further reacted into D_3_-XN-C and D_3_-IXN-C using a known selective cyclisation of the prenyl-group (10).

To overwhelm the electronically activation of the Michael system of XN, Stryker catalyst, which selectively lead to hydrogenation of the double bond, was used to synthesize the isotope labeled standard dihydroxanthohumol (DXN) by using a deuterated form rather than conventional donation of protons (Supplementary Material) (23, 34).

### Mass Spectrometry of Synthesized Isotopes

**Figure 3:**
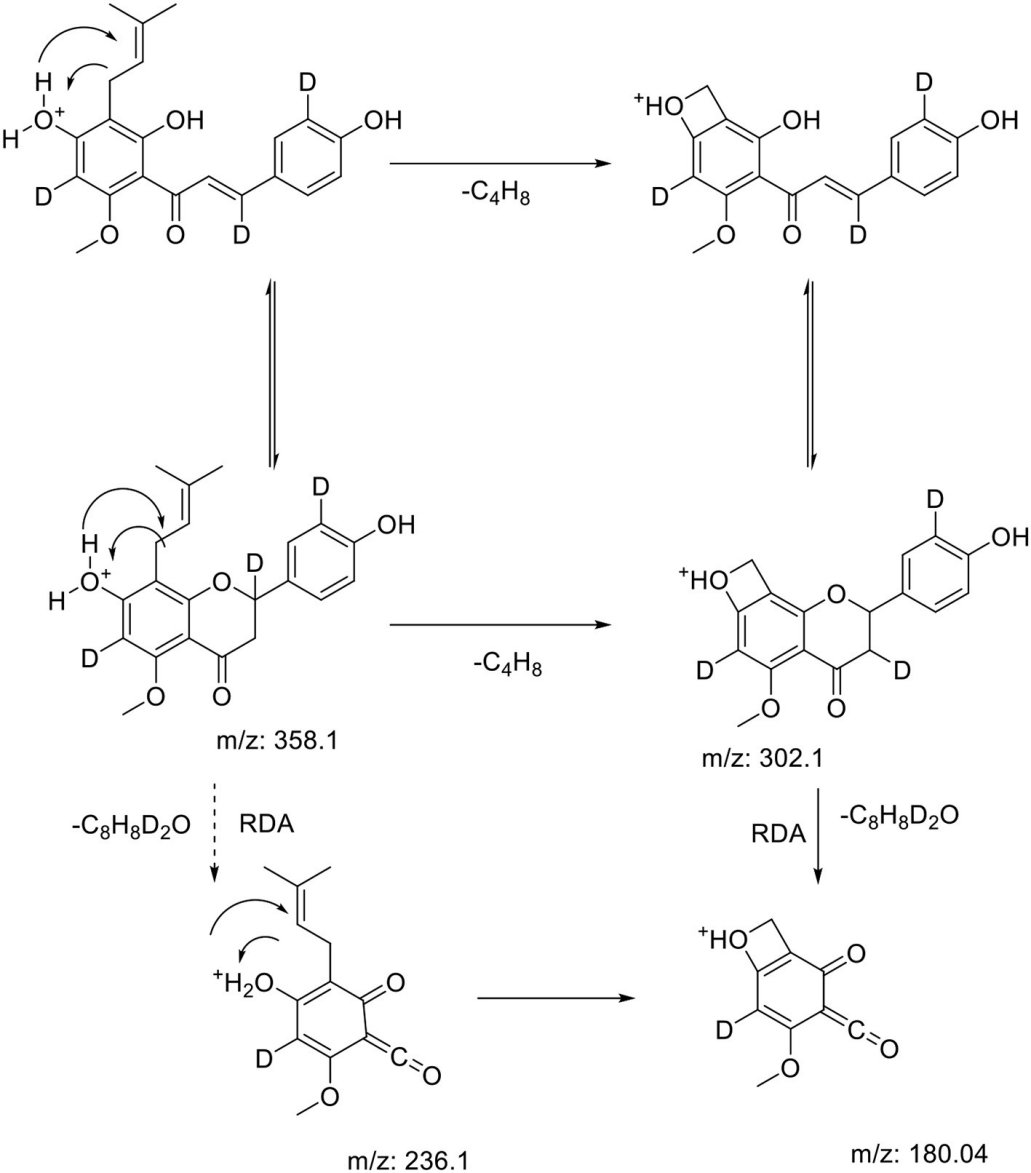
Fragmentation patterns of the prenylated flavonoids in hops adapted from Stevens et al. (35). The example given here is our proposed scheme using MRM information for the fragmentation of D_3_-XN. RDA is retro Diels Alder rearrangement. All fragments were observed during MRM of deuterated products.

The enrichment of deuterium in the isotopologues were confirmed using MS (see Table 1) and NMR analysis (Supplementary Material). In MS analysis, PFs fragmentate mainly via retro-diels alder which leads in case of the natural chalcone XN to the A-ring fragment (m/z 179) and the B-ring fragment (m/z 120). The fragmentation pattern of the isotopolog shows an A-ring (m/z 180), which leads to the assumption, that one deuterium is exchanged on the A-Ring. Furthermore, it can be concluded from fragmentation pattern, that one deuterium is exchanged on the B-Ring and one on the Michael system (Figure 3). Importantly no labeling is in the prenyl side chain as this would not be visible in MRM fragmentation and the fragment ion would have the same m/z as the precursor ion. A similar pattern can be seen in the analysis of D_3_-IXN, D_3_-6PN, and D_3_-8PN (Table 1). In case of the DXN derivative the high resolution mass spectrometry showed the incorporation of only one deuterium on the former α, β-unsaturated double bond (Supplementary Material), leading to the assumption that keto-enol equilibration does not save the deuterium in α-position from being exchanged. The NMR analyses confirmed the results of the mass spectrometry and are shown in the Supplementary Material.

The SIDA method should allow a simple, rapid and robust analysis concerning prenylflavonoids (PFs) for quantification in beer, hop products and future *in vivo* experiments. For the latter the DXN derivative is suitable, since the Michael system suggests a reduced bioavailability. All substances have a natural distribution of isotopes (in particular carbon-12 and carbon-13) and for an accurate SIDA there needs to be a certain increase in the mass making the difference measurable by mass spectrometer. The fact that DXN only has one D label made it not viable to be used in the SIDA method. However, the isotope analogs of the main natural prenylflavonoids D_3_-IXN, D_3_-IXN-C, D_3_-8-PN, D_3_-6PN, D_3_-XN, and D_3_-XN-C were deemed viable as they all had an increase between m/z of +3 to +4 Daltons. Table 1 shows a summary of the results for collisions energies and the mass transitions that were selected during the MRM optimizations and were incorporated into the LC-MS/MS method (35).

### Method Validation

Calibration for SIDA was performed by measuring mixtures of the reference standards and the internal standards at known concentrations. The response factor was obtained by linear regression plots after testing linearity according to (30). The calibration curves for each compound plotted are Area(ref)/Area(IS) over the n(ref)/n(nIS) and resulted from a triple injection (Supplementary Figures). The LOQ and LOD was calculated in Microsoft Excel (Microsoft Corporation, USA) according to the requirements of Vogelgesang and Hadrich, i.e., blank matrices were spiked with reference compounds and internal standards at known concentrations near an estimated LOD (29). The samples were worked up according to section Beer and analyzed in triplicate and concentrations of each analyte were compared to the theoretical values. The LOD was set at the 95% confidence level, where the maximum peak of a blank would be detected (29). The LOQ was set at four times the LOD. In a sense the LOQ and LOQ was a recovery test, but performed at an extreme. Therefore, additional recoveries, were tested at different concentrations of analytes and not above the LOQ. The recoveries were determined on a low (0.004–0.009 mg/L) and a high level (0.025–4.0 mg/L) resulting in 81.0–113.3% (see Table 2) for each specific analyte.

**Table 2:** The LOD, LOQ recovery and precision determined in the hop free beer matrix of each analyte quantified using the developed SIDA method.

**Analyte**	**LOD μg/L**	**LOQ μg/L**	**Recovery% High 2-points (*n* = 3)**	**Recovery % Low 2-points (*n* = 3)**	**Intra-day Precision RSD% (*n* = 3)**	**Inter-day Precision RSD% (*n* = 3)**	**Injection Precision RSD% (*n* = 9)**
IXN	0.07	0.30	89.4–105	95.0–104	3.3	6.0	0.51
IXN-C	0.21	0.84	89.9–102	93.6–113	4.1	4.5	4.2
8-PN	0.32	1.3	106–109	108–113	2.09	8.2	4.87
6-PN	0.94	3.74	89.7–104	91.0–101	1.52	3.12	3.42
XN	0.06	0.24	90.0–109	90.9–93.0	3.15	0.51	1.24
XN-C	0.19	0.78	82.8–93.8	81.0–91.6	2.43	0.45	2.77

The LOD and LOQ (Table 2) are similar to previously reported values, although the levels for XN and IXN presented here are lower. Stevens et al., using a triple quadrupole LC-MS/MS system with selective reaction monitoring, reached a LOQ of 10 ng/mL, which is at least double of the values measured here (LOQ 0.3–3.74 ng/mL) (28). The improvement is probably due to the use of SIDA with the carrier effect using internal standards eluting simultaneously with the analytes (28). However, using the more conventional method, i.e., comparing the signal to noise ratio, might provide a lower LOD than the method of Vogelgesang, and Hadrich, as it has less stringent criteria by not spiking blank matrixes and reporting the response (29). Using a single quadrupole MS PFs were analyzed in urine with LODs between 0.2 and 0.6 ng/mL (36). Additionally, in the method described here only 1 μL of sample was injected into the MS and therefore, room for improvement might be possible as increasing the injection volume might increase the sensitivity. Perhaps for the analysis of clinical samples this would be necessary.

### Quantification of Hop Prenylated Flavonoids in Samples Using SIDA

For SIDA a simple LLE method was developed. It enabled a faster work up than solid phase extraction (SPE). Furthermore, it has been reported that LLE is superior in regards to retaining the PF as SPE results in poor recoveries for some analytes as each PF is eluted at different retention factors (1). This was also observed by us using SPE in the development of the analytical method. Spiking LLE samples with labeled standards allowed minimal clean-up steps and less losses of analytes due to the ratio of each analyte against the internal standard remaining constant throughout the work-up process as the physical properties of the internal standard and the analyte are virtually identical. Therefore, regardless of the total losses of analyte the concentration can be calculated as long as the analyte and the internal standard ratio is detectable. The matrix effect during beer analysis was anticipated by utilizing SIDA due to the respective labeled internal standards being eluted at the same time. A structurally different internal standard has a different elution time than the analyte, which can lead to large errors as there is no compensation for the matrix suppression or enhancement that may occur. Additionally, here for the first time we report the quantitation of IXN-C and XN-C in beer, which are relatively unstudied prenylated flavonoids. Lastly, it is the first SIDA method that has been utilized on prenylated flavonoids found in hops.

### LC-Parameters

**Figure 4:**
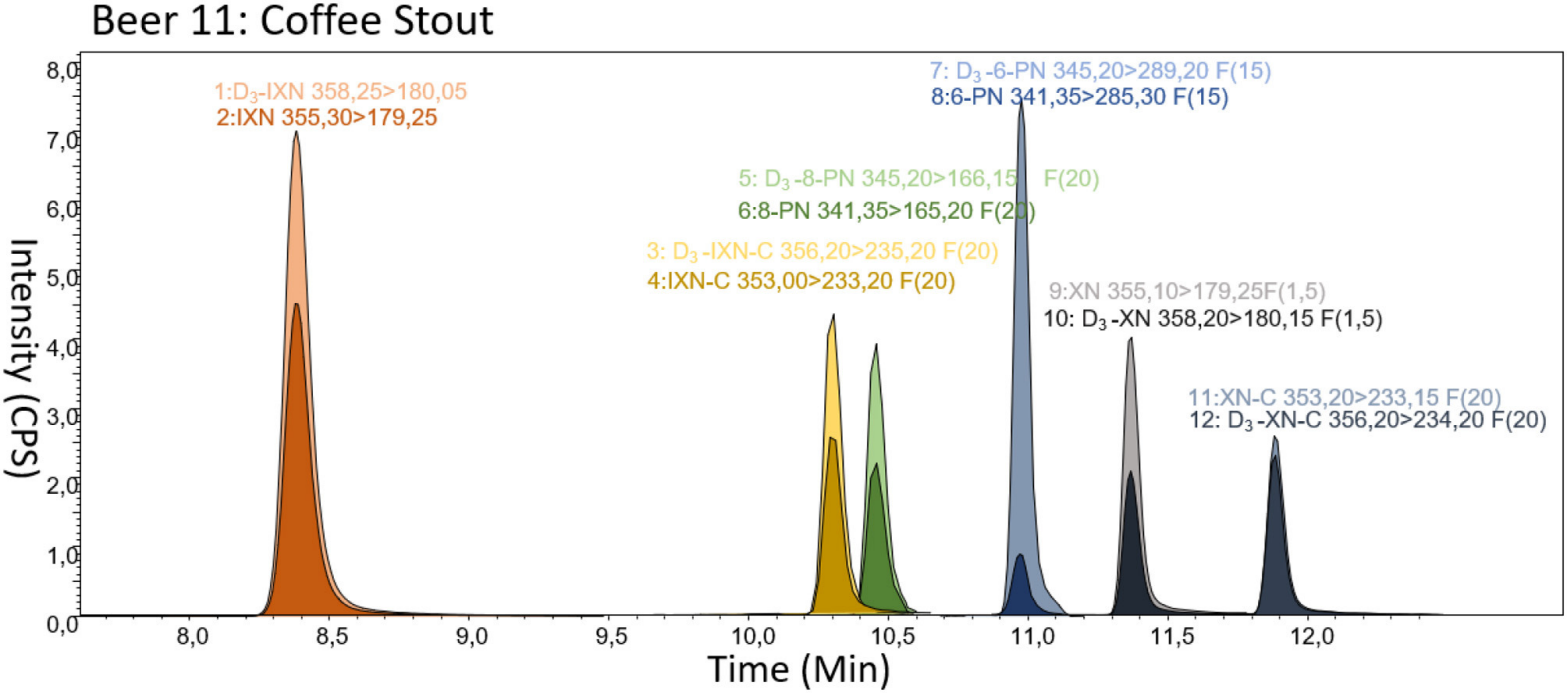
LC-MS/MS analysis of a beer spiked with the isotopically labeled standards. The spectra show the separation of all analytes along with selected fragmentation for qualifier and quantifier. F is the factor increased for each peak size relative to D_3_-IXN e.g., D_3_-IXN-C peak is increased 20 fold as it reveals significantly lower amounts in beer.

The LC method was designed so that sugars and carbohydrates that may have been extracted during the LLE were mostly omitted by diverting the initial 6 min of LC to waste. Additionally, the elution of the compounds of interest was devised to the isocratic phase of the method enabling a more uniform peak shape. The PFPP LC column used has been reported by Sus et al. (37) to be very suitable to separate prenylated flavonoids due to the phenolic-phenolic interactions (37). Additionally, compared to the C_18_ phase, no apparent retention time shifts were observed with the PFPP stationary phase (38). Thus, most of the analytes are eluted separately and IXN-C and 8-PN are almost base line separated (Figure 4) which is not a problem in MS quantification.

### Quantification of Prenylated Flavonoids in Beer, Hop Tea, and Hops

In order to prove the applicability of the new SIDA we quantified the PFs in different beers as summarized in Table 3. As the selection of beer samples are neither representative nor comprehensive, our results may only indicate the principle distribution of PFs in the different beer types. The total concentrations of PFs reported by (28) were between 43 and 4000 μg/L, and in European beers between 43 and 2680 μg/L (28, 39). Here we report comparable results between 723 and 2456 μg/L (see Table 3). A more recent analysis of beer polyphenols revealed a range of prenylated flavonoids of 0 and 9500 μg/L and highest containing beers were stouts and IPAs with an average range between 1.900 and of 2190 μg/L (40). The values of the stouts in general are comparable to our values (2445 μg/L), and only one sample (a black IPA with 9500 μg/L PF) which was not analyzed in our study, contained vastly more PF. It is worth mentioning that the alcohol free beer has a significantly reduced prenylated flavonoids content (1350 μg/L) compared to the IPA with alcohol (2400 μg/L). Obviously, the process of removing alcohol from beer seems to remove total PF content and reduce the amounts of compounds in the chalcone form, which are considered as desirable.

**Table 3:** Concentration of prenylated flavonoids found in 15 beer styles using the developed SIDA method: ± (SD μg/L, *n* = 3).

	**IXN μg/L**	**IXN-C μg/L**	**8-PN μg/L**	**6-PN μg/L**	**XN μg/L**	**XN-C μg/L**	**Total μg/L**
1. Helles (pale lager)	1160 (36)	12.0 (0.73)	19.7 (2.29)	46.7 (0.4)	19.8 (1.7)	1.9 (0.11)	1260
2. Festbier (festival beer)	1110 (26)	25.6 (6.30)	25.3 (1.14)	51.8 (0.90)	19.2 (2.5)	2.3 (0.09)	1234
3. Lager	627 (2.5)	13.7 (0.22)	14.4 (0.58)	14.2 (0.1)	22.1 (1.4)	1.9 (0.10)	693
4. Pilsner beer	989 (4.8)	20.5 (0.74)	7.0 (0.28)	22.8 (0.2)	10.1 (0.7)	1.8 (0.07)	1051
5. Hopped wheat beer A	1260 (53)	28.7 (0.74)	42.0 (1.07)	278 (5.6)	425 (35.1)	8.3 (0.45)	2042
6. Hopped wheat beer B	1400 (93)	15.4 (1.07)	36.5 (1.20)	120 (6.7)	411 (18.2)	10.2 (0.69)	1993
7. Double IPA	1320 (14)	12.8 (1.08)	50.6 (2.24)	421 (9.7)	500 (22.5)	16.1 (1.19)	2331
8. IPA	1540 (280)	14.1 (0.24)	54.6 (2.59)	368 (15.9)	464 (49.4)	5.1 (0.39)	2446
9. IPA alcohol free	1150 (147)	21.7 (4.96)	31.5 (1.98)	104 (3.2)	21.7 (3.9)	2.9 (0.37)	1332
10. Dunkel (dark beer)	1170 (128)	14.9 (1.35)	101.0 (7.55)	355 (3.1)	728 (30.0)	5.6 (0.20)	2375
11. Coffee stout	1480 (51)	23.4 (0.47)	88.7 (4.12)	351 (11.2)	483 (4.8)	18.8 (0.79)	2445
12. Bock beer	1550 (39)	19.0 (0.69)	50.3 (2.49)	156 (3.0)	99.3 (4.3)	4.4 (0.50)	1879
13. Doppelbock beer A	915 (15)	17.3 (1.75)	47.7 (2.64)	203 (2.40)	155 (29.9)	7.6 (0.09)	1346
14. Doppelbock beer B	1460 (80.6)	25.4 (1.67)	35.5 (1.45)	63.6 (0.6)	27.5 (1.8)	2.1 (0.25)	1614
15. Wheat bock beer	579 (13.9)	15.5 (1.00)	22.5 (1.12)	91.2 (3.2)	59.5 (3.9)	3.2 (0.20)	771

The amount of XN-C and IXN-C is very low in all beer samples, but it is shown that IXN-C is higher than XN-C in all beers measured, except for the double IPA. This is not surprising, since most of the XN is also converted to IXN during the brewing process (41). Interestingly, the amount of XN-C in the coffee stout is similar to the amount of IXN-C, which could suggest a stabilization process. This could be due to Maillard reaction products, which are supposed to stabilize XN from cyclizing in dark beer (42, 43). The double IPA is a heavily dry hopped beer and that might explain the higher XN-C amounts compared to IXN-C as the hops are not boiled to the extent of other beers. The chroman-like flavonoids XN-C and IXN-C are relatively unstudied and might confirm that they may add to the hepatoprotective properties that are said to outweigh the ethanol effects from beer consumption (44). However, this would need further research as little is known regarding the bioactivities of XN-C and IXN-C right now.

Furthermore, the concentrations of PFs in hop tea was investigated. However, the concentrations are so minimal that it would be very surprising if a health effect or taste modulation occurs (Table 4) (45). Drinking a tea that is brewed would only have 6.5 μg PF (based on 300 mL). Comparing this to the consumption of an alcoholic free beer which would contain 440 μg is vastly different. One optimistic observation is that IXN concentrations in brewed tea is not increased, which might imply that using hot distilled water does not cause cyclisation to the less desired IXN, contrary to boiling hops, a process essential in the manufacture of beer, where most XN is thermally cyclized into IXN (41). Perhaps a formulation of hop tea comprised from hop pellets or XN extract might be an alternative supplement as they contain very high concentrations of prenylated flavonoids of 11.200 mg/kg reported here with the main compound being XN (6640). Although the solubility of XN in hot water is not high (46, 47). However, the solubilization of other (e.g., IXN, 6PN, 8PN) PF at room temperature in water is higher, which might make tea formulations of hops a viable, but extremely bitter tasting dietary source (47).

**Table 4:** The concentration of the prenylated flavonoids in hops and hop tea ± (SD μg/L).

	**IXN**	**IXN-C**	**8-PN**	**6-PN**	**XN**	**XN-C**	**Total**
Hop pellets Columbus, μg/kg	4250	151	4.46	75.5	6640	76.9	11,200
15.6% α-acid (n=2)	(274)	(1.86)	(0.16)	(11.4)	(78.2)	(1.54)	
Hop tea (μg/bag) (n=2)	14.4 (2.44)	28.1 (0.52)	5.93 (0.52)	< LOD	17.9 (0.49)	3.12 (0.13)	69.0
Hop tea brewed (μg/L) (n=3)	3.4 (0.110)	12.4 (0.81)	1.58 (0.06)	< LOD	3.46 (0.49)	< LOD	20.8

## Concluding Remarks

The synthesis of the deuterated labeled internal standards applicable for prenylated flavonoids analysis has been developed using three different methods. Firstly using a microwave assisted demethylation while simultaneously enriching compounds with deuterium was envisaged and applied. Second, utilizing functional groups such as a Michael system and Strykers Cat incorporating deuterium and lastly, we have also shown that a method does not need a microwave reaction, which some laboratories could be limited by a lack of resources. By contrast, acid and base reactions are also suitable to introduce deuterium. Using the synthesized products we conducted a robust SIDA method for the analysis of six different PF found in beer and hops with comparable results (723–2456 μg/L) to other investigations. For a statistical differentiation of the beer types a broader and more comprehensive sampling would be necessary. Regarding the investigation of the PF content in hop tea it contains extremely low concentrations of PF, i.e., it is very unlikely the content is contributing to health or taste (45). The method here uses a fast clean-up of LLE of samples containing PF and compensates for losses during workup by the addition of isotopically labeled internal standards. The LOD and LOQ are excellent and precision is within the DIN 32645 guidelines for analytical methods of trace analysis (31). In future applications this method is applicable to measuring food samples, for freshness, food fraud and due to SIDA could be applied to biological samples as well. The validation results support that deuteration of PF is an easy pathway to producing labeled PFs and apply them to SIDA in quantifying PF in beer, hop tea and hops. The ongoing research regarding the bioactivities of these compounds provides a need for further analytical methods to be developed. The next step would be applying these methods to future clinical trial samples, such as blood, feces, and urine after consumption of beer or supplements. Reports of hop PF are already confirmed by urine analysis for biomarkers or in animal studies although SIDA is yet to be utilized (12, 48).

## Data Availability Statement

The raw data supporting the conclusions of this article will be made available by the authors, without undue reservation.

## Author Contributions

LB and CU wrote the manuscript and did the MS and NMR analysis of compounds synthesized. LB and MR designed the experiments for the microwave synthesis of the labeled XN, 6-PN, 8-PN, IXNC and XNC. LB carried out the synthetic work for all analytes excluding the stryker cat reaction of XN. LB and SS carried out the SIDA analysis including interpretation of the data. LB, SS, and CU carried out the preparative HPLC. SS performed the HPLC calibration graphs of XN-C and 8-PN. HR and CU designed the stryker cat synthesis of the xanthohumol and carried out the synthetic work for the DXN. All authors contributed to reading the article and proofing it before publication.

## Conflict of Interest

The authors declare that the research was conducted in the absence of any commercial or financial relationships that could be construed as a potential conflict of interest.
