# Supplementary material for: Stable Isotope Dilution Analysis of the Major Prenylated Flavonoids Found in Beer, Hop Tea, and Hops
Source: Front Nutr. 2020 Dec 15;7:619921. doi: 10.3389/fnut.2020.619921 (PMC7770140; doi:10.3389/fnut.2020.619921)
Supplement: Supplementary file 1 [file Table_1.DOCX]

Supplementary Material

# Synthesis of reference standards

## Xanthohumol

XN was produced from a CO_2_ hop extract waste of the cultivar Hallertau Taurus cultivar. To 100 g of the dry hop extract, 1,2 L of acetone (technical grade VWR) was added and stirred for approx. 1 h using a magnetic stir plate. The solvent extract was filtered with grade H 1/2 filter paper (Schleicher & Schuell) and evaporated leaving a dark green viscous extract (approx. 65 g). To the extract 50 mL of MeOH (VWR) was added along with 50 mL NaCl (0,2 M) solution causing a green precipitate (PPt) to form which was again filtered leaving an orange solution. The methanol was removed by rotation evaporation causing a further PPt to form, which was collected and subjected to flash chromatography by silica gel (mesh 70-230 mesh Sigma Aldrich, Deutschland) using 99:1 DCM:MeOH as an eluent. Fractions were checked for XN by TLC (Rf = 0.5) and pooled together resulting in an orange powder approx. 640 mg of XN. 1H NMR (400 MHz, MeOD) δ 7.80 (d, J = 15.5 Hz, 0H), 7.67 (d, J = 15.6 Hz, 0H), 7.50 (d, J = 8.6 Hz, 1H), 6.83 (d, J = 8.6 Hz, 1H), 6.02 (s, 0H), 5.27 – 5.09 (m, 1H), 3.90 (s, 1H), 3.23 (d, J = 7.1 Hz, 1H), 1.76 (s, 1H), 1.65 (s, 1H). 13C NMR (101 MHz, MeOD) δ 192.66, 164.74, 162.28, 161.00, 159.61, 141.88, 129.82, 127.06, 124.47, 122.84, 115.44, 107.99, 105.10, 90.22, 54.75, 47.60, 24.56, 20.88, 16.47.

## Isoxanthohumol

Argon was not utilised for this reaction. Using a method from Wihelm et al. 2006. XN (1 mM) was dissolved in 50 mL of a 1% NaOH (aq. w.v) (Wilhelm and Wessjohann, 2006). The reaction was on an ice bath and allowed to reach room temperature while stirring overnight. The solution was finally treated with 50% H_2_SO_4_ (aq. v.v) until the pH was neutralised causing a yellow PPt to form. Workup consisted of three times extraction using added EtOAc and a final wash of brine. The organic phase was dried using anhydrous Na_2_SO_4_ (VWR) and the product was subjected to Flash chromatography EtOAc:Pentane. 40:60% using silica gel (mesh 70-230 mesh Sigma Aldrich, Deutschland). Fractions containing IXN were check by TLC (Rf = 0.2) pooled and concentrated. Preparative HPLC (System 1) was used to purify. The reaction produced a 98% conversion according to HPLC, but only 34.8 mg was collected by preparative HPLC. ^1^H NMR (400 MHz, MeOD) δ 7.80 (d, *J* = 15.5 Hz, 1H), 7.67 (d, *J* = 15.6 Hz, 1H), 7.50 (d, *J* = 8.6 Hz, 2H), 6.83 (d, *J* = 8.6 Hz, 2H), 6.02 (s, 1H), 5.27 – 5.09 (m, 2H), 3.90 (s, 3H), 3.23 (d, *J* = 7.1 Hz, 3H), 1.76 (s, 4H), 1.65 (s, 3H).^13^C NMR (101 MHz, MeOD) δ 192.92, 164.27, 164.27, 163.83, 158.80, 131.67, 131.67, 128.86, 123.89, 116.24, 109.95, 109.95, 93.42, 79.99, 55.96, 46.21, 25.96, 22.70, 17.90.

## Isoxanthohumol-C

To 50 mg of XN 1% NaOH solution (20 mL) was added on ice. The reaction was left overnight and subsequently neutralised with drops of 50 % vv H_2_SO_4_ until a yellow PPt forms. The PPt was collected and dissolved in 50 mL of EtOAc and extracted 3-fold before the organic fraction was washed twice with water and a final brine solution. Na_2_SO_4_ was then used to dry the organic phase before the reaction was concentrated and directly dissolved in 5 mL of dry THF under argon atmosphere. To the reaction DDQ (64 mg) was added and the reaction was refluxed for 2 h. Subsequently, the addition of a 1% Na_2_S_2_O_3_ (2.5 mL) was used to quench the reaction. EtOAc (25 mL) was added to the reaction along with a further 2.5 mL of Na_2_S_2_O_3_. This was repeated 3 times until the organic phase turned yellow. The yellow organic phase was further washed with H_2_O (25 mL) 3 times and then finally with (25 mL) brine. The organic phase was further dried with Na_2_SO_4_, filtered, and concentrated before being dissolved in MeOH. The final preparation was purification using HPLC system 1. Yielding 7.62 mg. ^1^H NMR (400 MHz, MeOD) δ 7.38 – 7.27 (m, 2H), 6.86 – 6.76 (m, 2H), 6.52 (d, *J* = 10.2 Hz, 1H), 6.11 (s, 1H), 5.53 (d, *J* = 10.1 Hz, 1H), 5.36 (dd, *J* = 12.8, 2.9 Hz, 1H), 3.83 (s, 3H), 3.05 (dd, *J* = 16.6, 12.8 Hz, 1H), 2.71 (dd, *J* = 16.6, 3.0 Hz, 1H), 1.42 (d, *J* = 8.3 Hz, 6H). ^13^C NMR (101 MHz, MeOD) δ 192.34, 163.54, 161.96, 160.55, 159.00, 131.03, 128.89, 127.70, 116.74, 116.37, 106.34, 104.10, 94.71, 80.34, 56.37, 46.13, 28.38.

## Xanthohumol-C

An adaption to the method of Stevens et al. 1997 and Roeher et al 2018 (Stevens et al., 1997; Roehrer et al., 2018). XN (0,28 mM) was dissolved in 5 mL dry THF in a heated out double necked flask. The flask was left in an ice bath for 5 minutes before DDQ (0,28 mM) was added and the reaction turned black/green. The reaction was taken to reflux for 1 h before addition of a 1% Na_2_S_2_O_3_ (aq.) was added to quench the reaction. Aqueous work up included 3 times wash with Na_2_S_2_O_3_ in water (25 mL) and 25 mL of EtOAC: Followed by a further 3 times washing of the organic phase with H2O. The organic phase was collected and dried with Na2SO4 and evaporated before flash chromatography. The crude reaction was dry loaded and eluted using 20:80 EtOAc: hexane. The first compound that eluted was XN-C. Yield: 47%. Final clean-up was performed with HPLC System 1.1H NMR (400 MHz, CD3CN) δ 7.81 – 7.67 (m, 2H), 7.57 – 7.44 (m, 2H), 6.89 – 6.77 (m, 2H), 6.62 (d, J = 10.0 Hz, 1H), 6.03 (s, 1H), 5.53 (d, J = 10.0 Hz, 1H), 3.94 (s, 3H), 1.43 (s, 6H). 13C NMR (101 MHz, CD3CN) δ 194.28, 164.16, 163.02, 161.57, 161.27, 144.13, 131.42, 128.29, 126.69, 125.36, 116.91, 107.01, 103.93, 92.78, 79.17, 56.54, 28.59.

## 8-Prenlnaringenin and 6-Prenylnaringenin

An adaption from Urmann and Riepl 2020 and Wilhelm et al 2006. XN (1 mM) was added into a double necked flask along with LiCl (20 mM) and KI (2 mM) (Wilhelm and Wessjohann, 2006; Urmann and Riepl, 2020). Argon was added to protect the reaction along with 4 mL DMF. The reaction underwent reflux for 4 h and then was left at room temperature overnight. To quench the reaction 1,5 mL of HCl (25%) was added causing a yellow precipitate to form. EtOAc (25 mL) was added and the reaction was washed with Na_2_S_2_O_3_ (aq) and further washed with H_2_O 4 times 25 mL. Brine was used in the final stage and further dried using Na_2_SO_4_. The reaction was filtered and columned using silica gel 99:1 DCM:MeOH. The fractions that contained 8- and 6-PN were then separated using sephadex LH20 with methanol as an eluent. Yielding 0,2mM (20%) of a mixture of 6 and 8-PN this was separated using preparative HPLC. Using system 1. 6-PN. 8-PN. ^1^H NMR (400 MHz, MeOD) δ 7.32 (d, *J* = 8.6 Hz, 2H), 6.82 (d, *J* = 8.6 Hz, 2H), 5.93 (s, 1H), 5.32 (dd, *J* = 12.8, 3.0 Hz, 1H), 5.14 (t, *J* = 8.7, 5.9, 2.9, 1.5 Hz, 1H), 3.17 (d, *J* = 7.4 Hz, 2H), 3.08 (dd, *J* = 17.1, 12.8 Hz, 1H), 2.71 (dd, *J* = 17.1, 3.1 Hz, 1H), 1.62 (d, *J* = 1.4 Hz, 4H), 1.57 (d, *J* = 1.4 Hz, 3H). ^13^C NMR (101 MHz, MeOD) δ 198.17, 166.07, 163.14, 161.57, 158.89, 131.58, 131.39, 128.93, 123.93, 116.26, 109.03, 103.36, 96.37, 80.26, 49.00, 43.97, 25.96, 22.48, 17.88. 6-PN ^1^H NMR (400 MHz, MeOD) δ 7.35 – 7.28 (m, 2H), 6.81 (d, *J* = 8.6 Hz, 2H), 5.93 (s, 1H), 5.31 (dd, *J* = 13.0, 2.9 Hz, 1H), 5.19 (t, *J* = 7.2 Hz, 1H), 4.58 (s, 1H), 3.20 (d, *J* = 7.2 Hz, 2H), 3.10 (dd, *J* = 17.1, 13.0 Hz, 1H), 2.68 (dd, *J* = 17.1, 3.0 Hz, 1H), 1.75 (s, 3H), 1.65 (s, 3H). ^13^C NMR (101 MHz, MeOD) δ 197.85, 165.93, 162.56, 158.98, 131.59, 131.25, 128.99, 123.88, 116.30, 109.66, 103.19, 95.40, 80.45, 44.20, 25.94, 21.87, 17.84.

# Synthesis of Isotopically labelled standards

## D_3_-Xanthohumol, D_3_-Isoxanthohumol D_3_-6-Prenylnaringenin and D_3_-8-prenylnaringenin

To the extracted XN (50.3 mg) 5 mL of DMF and 5 mL D_2_O, approx. 1 mg Pd (30%)/C, approx. 1 mg Pt/C and approx. 1 mg Rh (10%)/Aluminium was added into an 80 mL quartz microwave reaction flask with a magnetic stir bar (CEM). Microwave (Discover SP, CEM) was set to react for 150°C, 2 min, 10 bar, 150 w with vigorous stirring set. After the reaction had cooled 138 mg of LiCl was added and the reaction allowed to stir for 5 min before being raised to 250°C, 10 min, 20 bar, 250 w. The reaction was cooled, and TLC (50:50 EtoAc: Pentane) showed that there were three spots (Rf 0.8, 0.5 0.2) had formed. Subsequently 80, µL of D_2_SO_4_ was added causing a precipitate to form. For workup 25 mL of EtOAc and 25 mL Na_2_S_2_O_3_ (aq) was used for the initial extraction followed by 2 times EtOAc: water extraction. The organic phase was dried using Na_2_SO_4_, filtered and evaporate. The sample was then dissolved in 3 mL of MeOH and prepared using preparative HPLC system 1. Yields: D_3_-XN 5.07 mg, D_3_-IXN 4.38 mg , D_3_-6-PN, 1.13 mg, D_3_-8-PN 0.4 mg (confirmed by HPLC).

ESI + 358.20. D_3_-XN ^1^H NMR (400 MHz, Methanol-*d*_4_) δ 7.66 (s, 1H), 7.50 (t, *J* = 8.0 Hz, 2H), 6.83 (d, *J* = 8.8 Hz, 1H), 5.20 (tt, *J* = 7.2, 1.4 Hz, 1H), 3.90 (s, 3H), 3.23 (d, *J* = 7.2 Hz, 2H), 1.76 (s, 3H), 1.65 (s, 3H). 13C NMR (101 MHz, MeOD) δ 194.01, 166.16, 163.74, 162.39, 161.04, 161.00, 131.35, 131.25 , 131.14, 130.28, 128.45, 116.88, 116.16, 109.40, 106.46, 91.66, 56.18, 49.64, 25.96, 22.27, 17.87.

ESI + 358.25., D_3_-IXN ^1^H NMR (400 MHz, Methanol-*d*_4_) δ 7.34 – 7.27 (d, *J* = 8.2 Hz, 2H), 6.84 – 6.77 (d, *J* = 8.2 Hz, 2H), 5.29 – 5.24 (s, 1H), 5.17 – 5.11 (t, 1H), 3.81 – 3.77 (s, 3H), 3.26 – 3.17 (d, *J* = 7.6 Hz, 2H), 1.63 – 1.60 (d, *J* = 1.4 Hz, 3H), 1.57 – 1.54 (d, *J* = 1.3 Hz, 3H). ESI+ 6-PN 345.20. 1H NMR (400 MHz, MeOD) δ 7.35 – 7.30 (m, 2H), 6.83 (d, J = 8.8 Hz, 1H), 5.32 (s, 1H), 5.18 (t, J = 7.2 Hz, 3H), 3.21 (d, J = 7.2 Hz, 21H), 3.17 – 3.08 (m, 2H), 2.73 – 2.62 (m, 2H), 1.76 (s, 3H), 1.65 (s, 3H). ESI + 8-PN 345.20 – due to the low amounts of 8-PN produced and a contaminant that was not able to be separated during preparative HPLC in the reaction no NMR was possible, although some information from the NMR could be deduced.

The ^1^H NMR spectra of D_3_-XN (Figure 1) has a total of three protons missing compared to that of XN when using the sum of integrals. The protons that are exchanged with deuterium are of 7.84-7.75 ppm (100%) of 6.86-6.79- (75%) exchanged of 6.05 ppm (91%) exchanged which is estimated by the lack of a signal (See Figure 3). The exchanged protons coincide with the MS data reported in the results section as the m/z is plus 3 protons. Additionally, further evidence is in the multiplicity of the NMR spectra allowing us to confirm that one of the Michael system is exchanged due to the multiplicity of the signal at 7.67 ppm (Proton in position 2) being a singlet compared a doublet in normal XN (Figure 1).


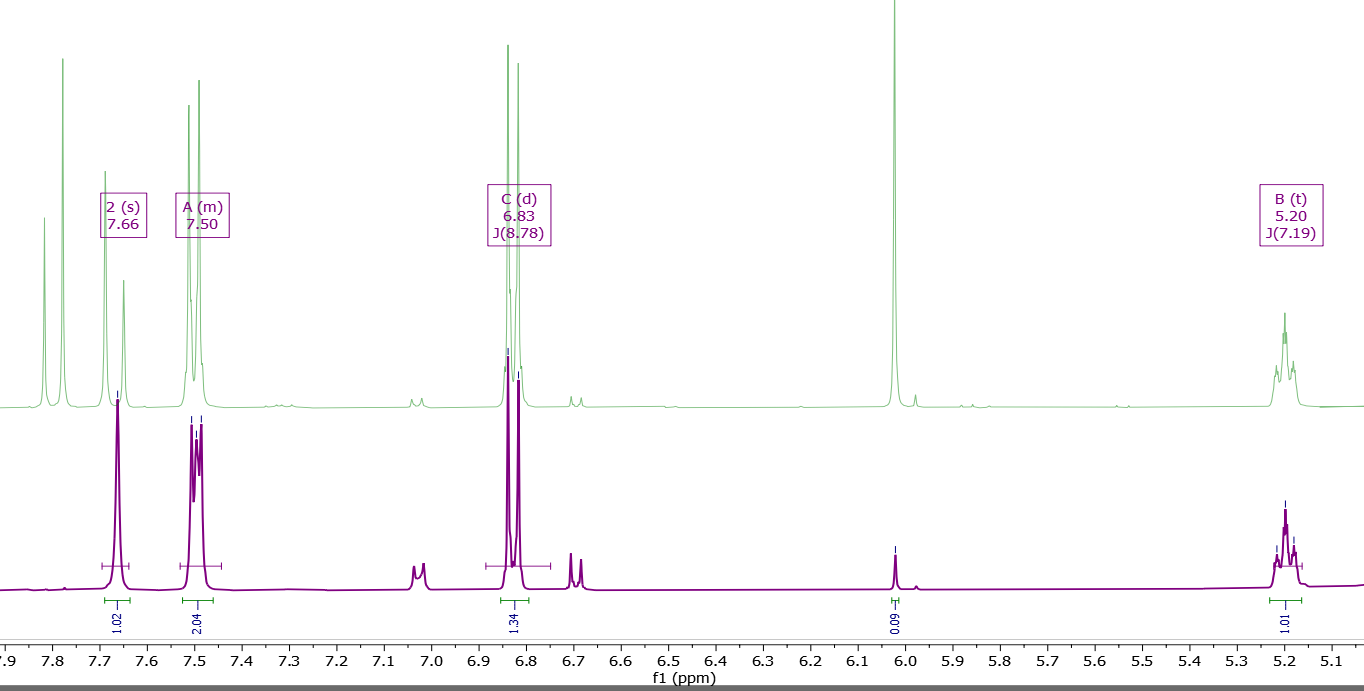


ppm

8

2‘

2

1

**Figure 1.** The superimposition of D_3_-XN (purple) with XN (green). The doublet of position 2 is gone and 3’ is reduced to 1 proton and position 8 is only visible for some residual signal from remaining proton.

The ESI + data of D_3_-IXN showed that there is a mass increase of +3. The NMR data showed which protons were exchanged. Figure 5, shows the spectrum of D_3_-IXN and due to the lack of the proton at 6.0 ppm we can confirm that the aromatic proton equivalent to position 8 in Figure 3 is exchanged. Similar to XN the doublet in position 1 after the deuteration is now a singlet. And at 3.75 ppm the proton see Figure 4. Therefore, it is similarly deuterated as the XN.


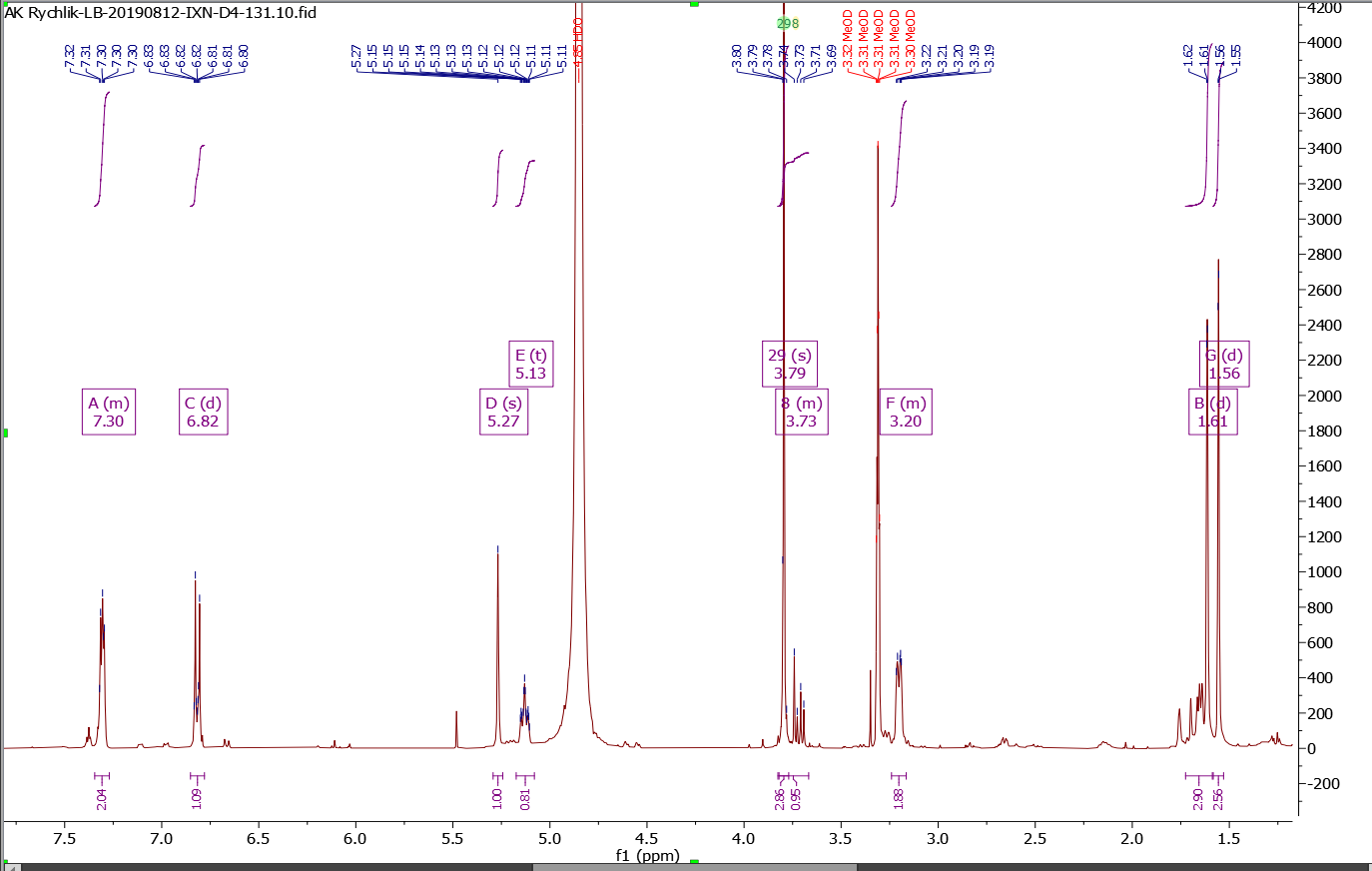


**Figure 2**: D_3_-IXN NMR spectra. The protons equivalent to the D3-XN are exchanged.

The ESI+ of D_3_-6-PN represents an m/z of +4: Therefore, the assumption is that 4 protons have been exchanged. The NMR shows that there are a different degree of labelling compared with XN. The NMR spectrum in Figure 5 demonstrates that no aliphatic proton was exchanged, although there is the similar degree of deuteration as D3-XN there is additional deuteration in the aromatic ring which is calculated by the integrals of each peak compared to non-deuterated 6-PN. Although, it is difficult to calculate the degree accurately due to the low concentration of the sample in the NMR. Hence, the degree of deuteration is closer to 4.

**Figure 3**: D_3_-6-PN (Red) superimposed with 6-PN (cyan). Position 8 is completely exchanged. Based on the integrals all aromatic have some degree of deuteration which collectively is near 4 from the MS results.

Similar to the D_3_-6-PN, D_3_-8-PN showed an m/z of +4: The D_3_-8-PN was produced in amounts that were not sufficient for 1H NMR and there was a contaminant at 8.09 min (see Figure 4) that could not be removed after several attempts of preparative HPLC. Therefore, to characterise the sample it underwent HPLC and MS analysis.


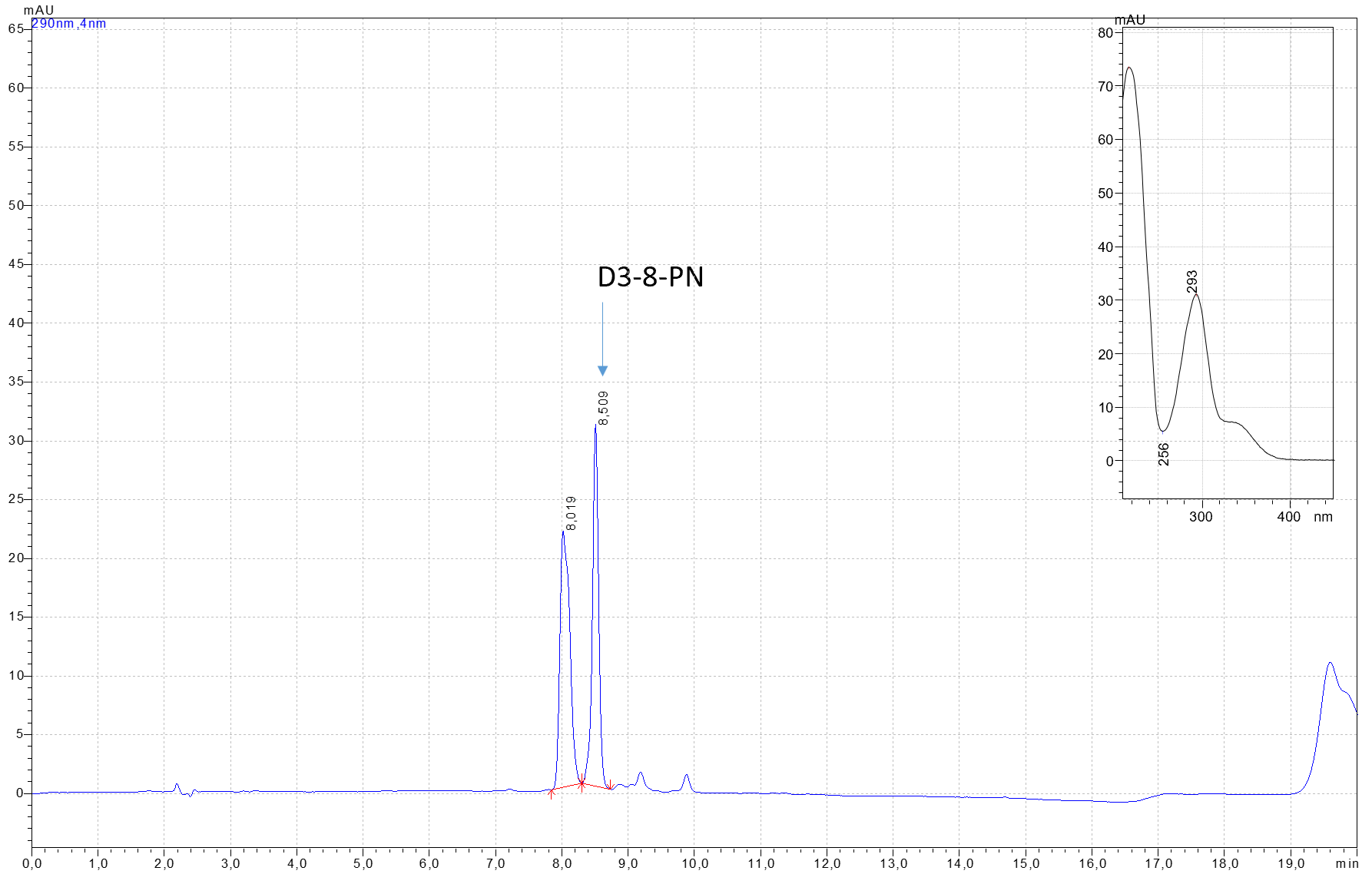


**Figure 4**: HPLC chromatogram of D_3_-8-PN and the contaminant. The retention time and the UV profile (top right) of D_3_-8-PN is the same as non-deuterated 8-PN.

## D_3_-Isoxanthohumol (non-microwave)

XN, 0.14 mM was dissolved in D_2_O with the addition of NaOD (250 µL). The reaction was left on an ice bath overnight and afterwards 84 µL of D_2_SO_4_ was added causing a yellow precipitate to form. The D_2_O was removed by evaporation, and further enrichment was achieved by the addition of fresh D_2_O and refluxed, along with a few mg of Pd/C 10%, for 6 h. The process was repeated. Work up was by the addition of EtOAc and water (DI) and the organic phase was collected the EtOAc was repeated three times until a final wash of the organic phase with brine. The organic phase was collected dried using Na_2_SO_4_ (anhydrous), filtered and evaporated. Columned using 99:1 DCM:MeOH. Fractions that contained d_3_ -IXN were collected. The final preparation utilised preparative HPLC system 2. Yield 98%. ESI+ 358.14. IXN ^1^H NMR (400 MHz, Methanol-*d*_4_) δ 7.31 (d, *J* = 8.5 Hz, 7H), 6.81 (d, *J* = 8.6 Hz, 18H), 6.12 (s, 1H), 5.28 (dd, *J* = 12.8, 3.0 Hz, 2H), 5.14 (t, 1H), 3.80 (s, 4H), 3.21 (d, *J* = 6.0 Hz, 2H), 2.98 (dd, *J* = 16.7, 12.8 Hz, 1 ) 2.71 – 2.60 (m, 2H), 1.62 (s, 4H), 1.56 (s, 5H). ^13^C NMR (101 MHz, Methanol-*d*_4_) δ, 192.92, 164.27, 163.83, 158.80, 131.67, 131.67, 128.86, 123.89, 116.24, 109.95, 109.95, 93.42, 79.99, 55.96, 46.21, 25.96, 22.70, 17.90.

## D_3_-Isoxanthohumol-C and D_3_-Xanthohumol-C

To prepare D_3_-IXN-C, XN was treated the same as section 2.3.2 and then further treated by dissolving D_3_-IXN 20 mg in 5 mL THF followed by the addition of 36 mg of DDQ. The reaction was taken to reflux and left to react for 4h before being quenched by the addition of 2.5 mL a 1% Na_2_SO_4_ solution. Workup included 3 times wash with H_2_O: EtoAC 50:50 and the H_2_O fraction washed 3 times with equal amounts of EtOAc. The organic phase was collected and dried with Na_2_SO_4_ and evaporated before flash chromatography. The crude reaction was dry loaded and eluted using 20:80 EtOAc: pentane. XN-C was prepared the same way as d_3_-IXN-C although d_3_-XN (4 mg) was the starting material. Preparative HPLC system 1 was used in the final clean-up of both compounds. Yielding, d_3_-IXN-C 4.76 and d3-XN-C 1.13 mg. D_3_-IXNC NMR ESI^+^ (356.15) ^1^H NMR (400 MHz, Methanol-*d*_4_) δ 7.32 (d, *J* = 8.5 Hz, 1H), 6.83 (d, *J* = 8.6 Hz, 1H), 6.53 (d, *J* = 10.1 Hz, 1H), 6.11 (s, 0H), 5.54 (d, *J* = 10.1 Hz, 0H), 5.36 (s, 0H), 3.83 (s, 1H), 1.43 (s, 1H), 1.41 (s, 1H). ^13^C NMR (101 MHz, Methanol-*d*_4_) δ 28.37 (4'', 5''), 28.66, 45.44 (3), 56.38 (OCH3), 79.19 (3''), 80.26 (2), 94.70 (6), 104.12 (8), 106.35 (4a), 116.38 (5', 3'), 116.74 (1''), 127.72 (2''), 128.91 (6', 2'), 131.04 (1'), 159.01 (4'), 160.56 (7, 8a), 161.95, 163.51 (5), 192.45 (4). D_3_-XN-C ESI + 356.20 ^1^H NMR (500 MHz, MeOD) δ 7.71 (d, *J* = 7.9 Hz, 1H), 7.52 (q, *J* = 4.1 Hz, 2H), 6.83 (d, *J* = 8.8 Hz, 1H), 4.88 (s, 1H), 3.93 (d, *J* = 20.8 Hz, 3H), 3.35 (s, 5H), 1.34 (s, 6H).

D_3_-IXN-C was produced from D_3_-IXN (non-microwaved) by the selective prenyl cyclisation using DDQ, a known reaction that was initially carried out by Mahoney et al. (Mahoney et al., 1988)Upon preparative HPLC there were 2 major product, which one was D_3_-IXN-C. The same method was applied to XN using the same molar equivalent and resulted in D_3_-XN-C. Figure 8, shows the protons that were exchanged which is the equivalent to non-microwave D_3_-IXN, therefore the compounds are stable enough to perform harsh reactions and retaining the deuteration.

**Figure 5**: D-3-IXN-C (red) that was synthesised from non-microwave D3-IXN superimposed with IXN-C (Cyan).

The exact same procedure using the same molar equivalent of DDQ was applied to a proportion of the D_3_-XN (See section 2.3.3), the results show that the equivalent positions were deuterated. Position 2, 8 and 15 (See Figure 9).

**Figure 6**: XN-C (cyan) superimposed with D3-XN-C (red). The assignments are for XN-C and therefore, it is possible to see which protons are exchanged. Due the lack of a peak (position 8 and 2).

The NMR (Figure 10) shows that the deuterium exchange using a non-microwave approach has a different deuteration pattern than with microwave assistance. First, using the NaOD and D_2_SO4 causes both protons at position 2 to be exchanged. The structure of the non-microwaved acid/base catalysed reaction that formed D_3_-IXN. There was partial deuterium exchange for proton 8 which can explain the m/z of 358.14 value observed during MS analysis. Although upon attempts to demethylate the D_3_-IXN to produce D3-8PN were unsuccessful the cyclisation of the prenyl group was achieved as mentioned before.

1.

2.

8.

**Figure 7:** D3-IXN (Red) superimposed with IXN (Cyan). The protons that are exchanged are in position 2. Take not the position 1 is now a singlet indicating the complete conversion of the protons as in IXN this is a double indicating the proximity to two protons.

## Isotope labelled DXN (xanthohumol-derivative without Michael system)

Preparation of DXN

a) Ph_2_SiD_2_

5 g dichlordiphenylsilane was dissolved in 30 ml THF (rotisolv) in a three necked reaction vessel equipped with a nitrogen inlet, condenser and relief vent. 640 mg LiD was added and the suspension is refluxed under nitrogen for 48 h. After cooling, the suspension is filtered through a glass frit, filled with a dense bed of celite (2 cm). The clear filtrate is stripped from THF by soft application of vacuum and carefully distilled under nitrogen, using a short-path vacuum distillation apparatus (“balltube”)(Büchi Mod.).

b) Stryker catalyst

To prepare the Stryker catalyst, the method according to Lee is followed, with the exception of using Ph_2_SiD_2_.(Lee and Yun, 2005)

c) DXN

Afterwards, 44 mg deuterated Stryker catalyst was suspended in 6 ml dry benzene in a Schlenk tube under nitrogen atmosphere. 390 mg Ph_2_SiD_2_ was added and after 10 min stirring, 100 mg xanthohumol was added. After stirring over night at room temperature, 5 ml D_2_O was added followed by 15 ml EtOAc. The aqueous phase was separated and the organic phase was mixed with 2 g silica gel G60. The solvent was removed and the resulting dry load mixture was subjected to the top of a small column of silica gel G60 (length 2 cm, diameter 1 cm) and eluted with EtOAc, 75 ml. This crude fraction containing also phosphane and some silane decomposition products was chromatographically purified. After removing the solvent, the residue was dissolved in 5 mL methanol and filtrated over glass wool. Methanol was removed under nitrogen stream, the residue was dissolved in acetonitrile/water (2/3) and the cleaning procedure started using solid phase extraction with a Hypersep C_18_ column (1000 mg 6 mL; Thermo). The product was eluted using acetonitrile/water 35/65 and these fractions were lyophilized to remove the solvent. The residue was then purified using preparative HPLC (System 4). After lyophilization of the product fractions, the product was obtained as a white powder (7.2 mg). HRMS-ain positive ionization mode(IT TOF; Shimadzu): [M+H]^+^ 358.1761 (predicted 358.1759; Δ = 0.2mDa; Δ = 0.56 ppm) for the sum formula of C_21_H_23_DO_5_. D_2_-XN ^1^H NMR (400 MHz, Methanol-*d*_4_) 7.02 (d, *J* = 8.3 Hz, 2H), 6.685 (d, *J* = 8.7 Hz, 2H), 5.966 (s, 1H), 5.159 (t, J=7.3Hz), 3.811(s, 3H), 3.183 (m, *J* = 7.8 Hz, J=7.33Hz, 3H), 2.814 (t, J=7.8 Hz, 1H), 1.737 (s, 3H), 1.634 (s, 3H) . ^13^C NMR (400 MHz, MeOD) δ 206.5, 165.76, 163.81, 162.64, 156.52, 133.88, 131.31, 130.29, 124.26, 116.15, 109.25, 105.76, 91.24, 55.88, 47.46, 25.95, 22.21, 17.86, 14.42.

# Preparative and analytical HPLC

### Preparative HPLC System 1

The preparative HPLC was carried out using a YMC-ACTUS Tri art column (Triart C18, 150 x 20.0 mm, l.D. S-5 µm, 12 nm) connected with YMC-Guardpack Pro (C18, 30 x 10.0 mml.D. S-5 µm, 12 nm) guard column. The HPLC system comprised of a BESTA pump system (Besta Type HD 2-400 pump, Wilhelmsfeld, Germany) coupled to a Sapphire variable wavelength detector (Ecom, Prague, Czech Republic). The wavelength was set to 290 nm for the initial at 0-15.5 min and at 370 nm afterwards. Samples was manually injected using a glass syringe (1 mL) and a valve (brand) initiated the set time program. The flow was set at 7.5 mL/min with HPLC grade H_2_O (VWR) and MeOH (VWR) starting with a multi-step gradient of 0 min 65 % MeOH, 0.5 min 80 % MeOH, 12.3 min 80 % MeOH, 12.6 min 85 % MeOH,16.5 min 85 % MeOH, 16.8 min 90 % MeOH, 24.5 min 90 % MeOH, 25.0 min 65 % MeOH, 30.0 min 65 % MeOH. All samples were manually collected.

### Preparative HPLC System 2

The preparative HPLC used the same as system 1 although starting with 80% MeOH and monitored only at 290 nm.

### Preparative HPLC System 3

D_3_-8-Prenylnaringenin underwent further purification due to overlapping peak with d_3_-IXN. The method was a modified version of system 1. Starting at 65% MeOH and remained for 7.5 min before being raised to 80% MeOH and left for isocratic until 20 mins. Lastly, solvent B was increased to 95% for 5 minutes which the column was re-equilibrated at 65% MeOH.

### Preparative HPLC system 4

To purify the isotope-labelled Xanthohumol derivative without Michael system the preperative HPLC-System Puriflash 250 (Interchim) and a C18-column (Phenomenex, Luna C18(2) 250x15 mm 5u) was used. The gradient used started with 40% A (acetonitrile) and 60% B (water) and raised to 95 % B within 45 minutes.

### Chromatograms of Prep HPLC

**
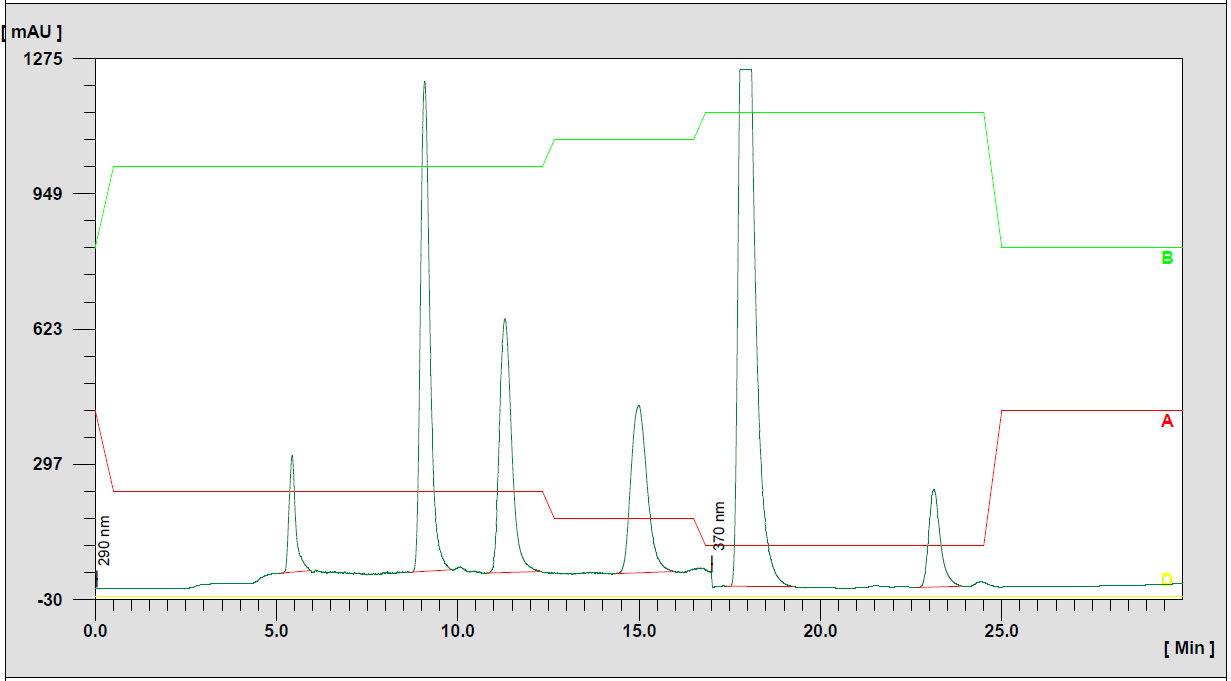
Figure 8**: Preparative HPLC of Reference compounds IXN (rt=9.4), 8PN (rt=11), 6PN (rt=15) and XN (rt= 19)

**
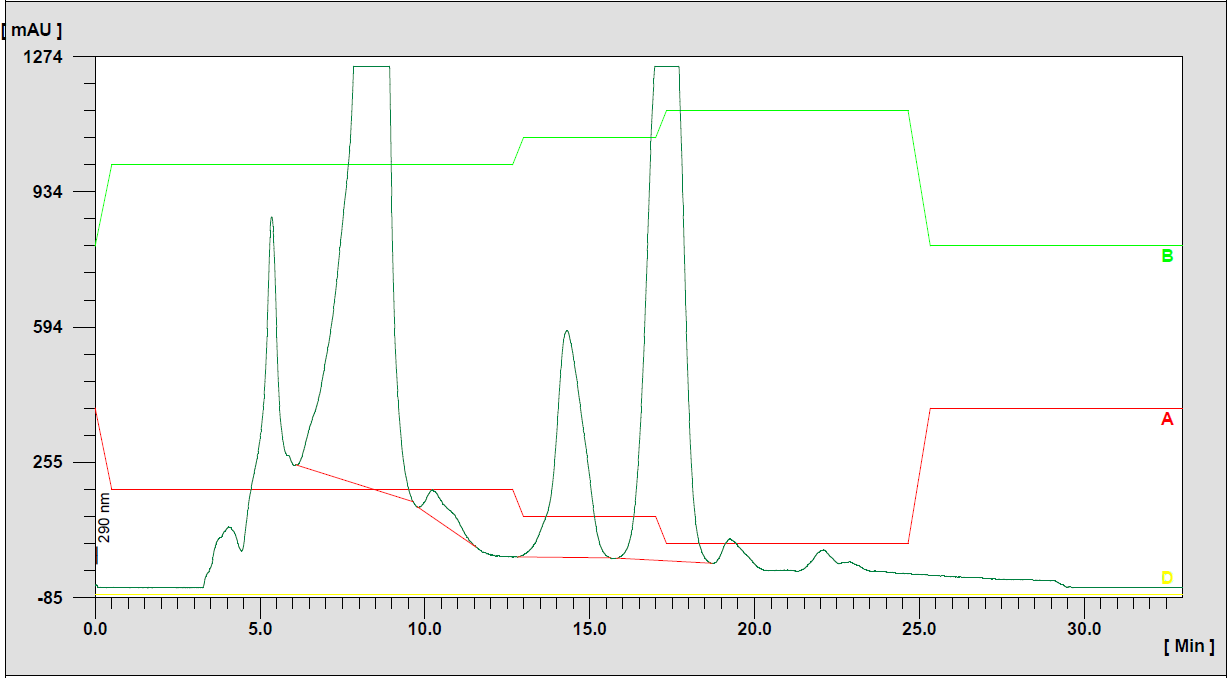
Figure 9:** Preparative HPLC of Deuterated isotopes, D_3_-IXN (rt = 9), D_3_-6-PN(rt=10), D_3_-8PN (rt =14.8) and D_3_-XN (rt=19).

**
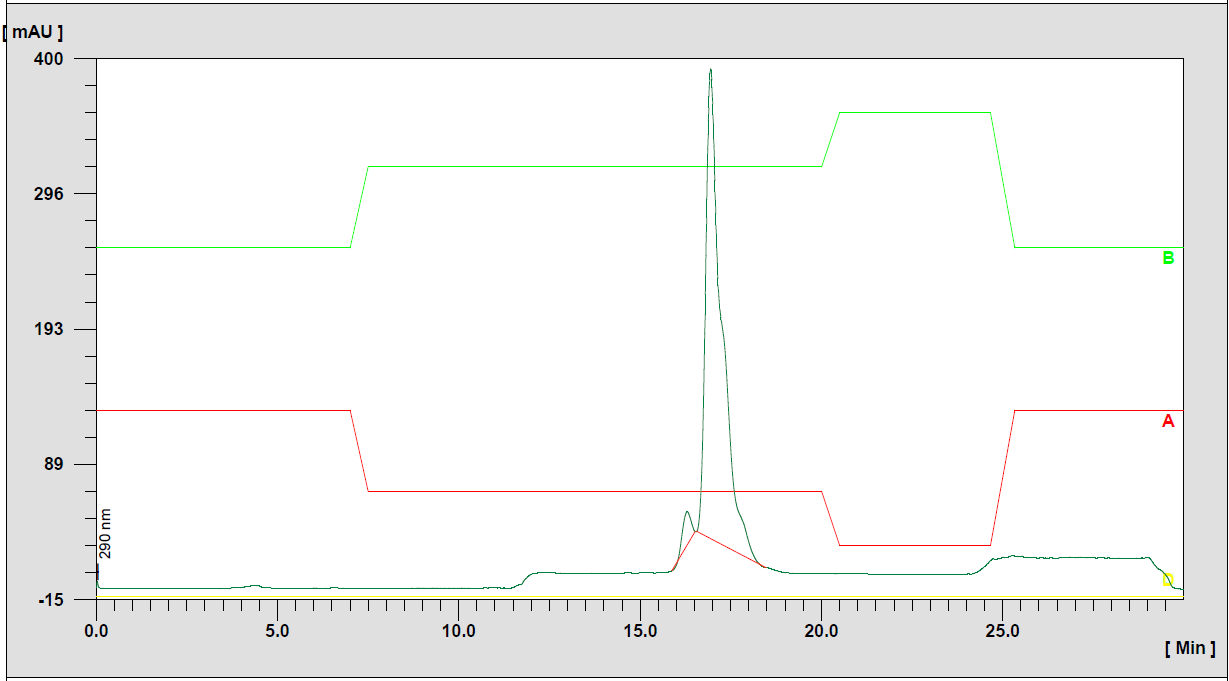
Figure 10**. Preparative HPLC of D_3_-8-PN after further clean up.

**
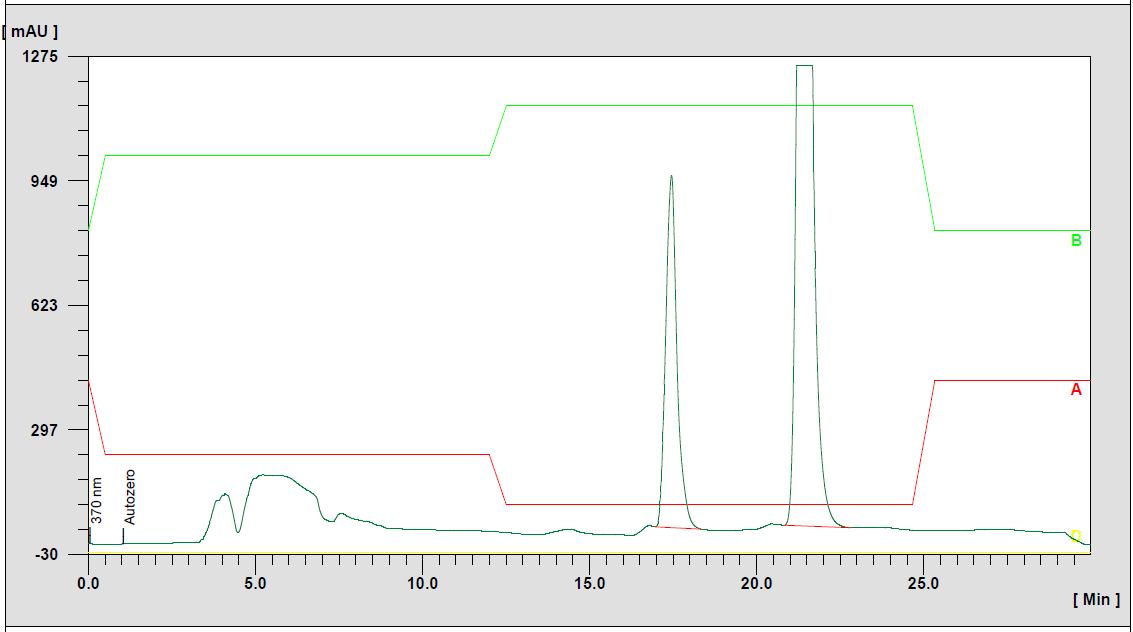
Figure 11**. Preparative HPLC of D_3_-XN -C (rt= 22).

**
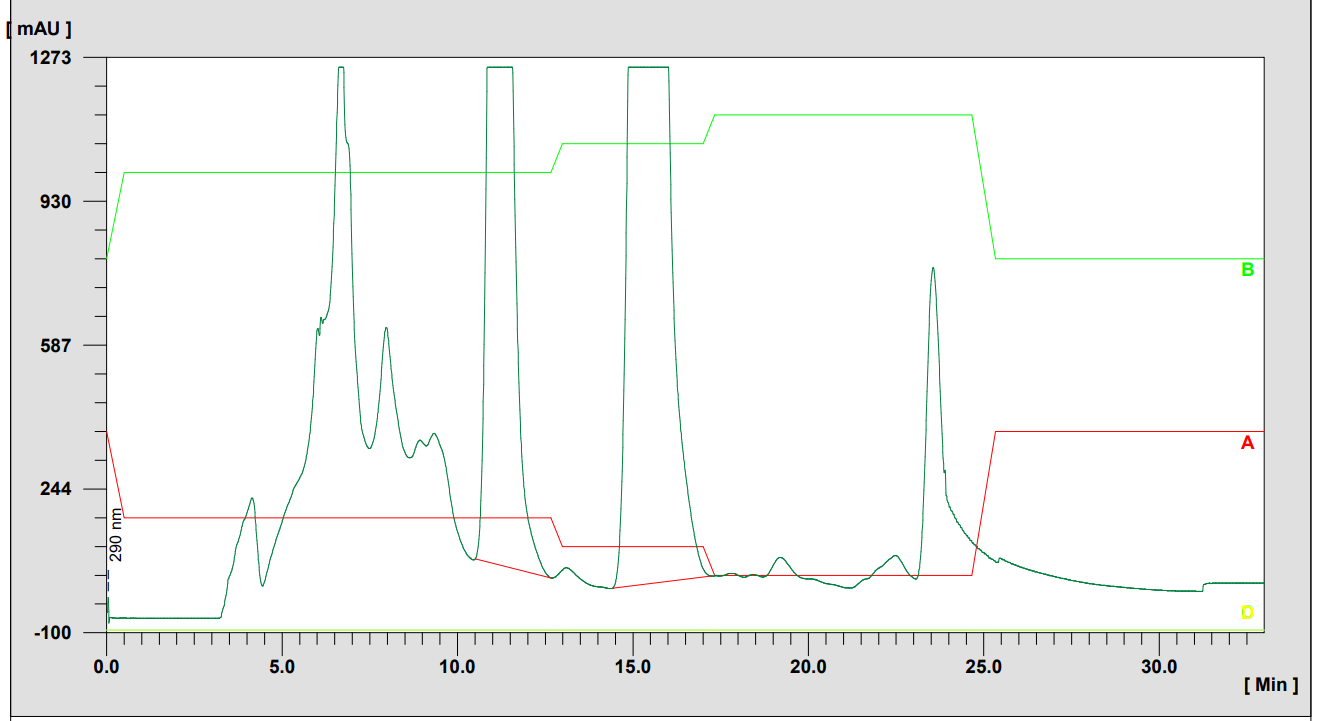
Figure 12**: Preparative HPLC of D_3_-IXN-C (rt = 15).

### Analytical HPLC

Due to the low amounts of D_3_-8-PN and D_3_-XN-C synthesized, HPLC was necessary to quantify. Calibration graphs (See supplementary information) were produced using standards of 8-PN and XN-C quantified using qNMR dissolved in ACN. The calibration graphs were used to produce linear regression equations and subsequently the concentrations of D_3_-8-PN and D_3_-XN-C quantified.

**Figure 14**: The calibration graph of 8-PN. D3-8-PN concentration was calculated using the above equation.

**Figure 15**: The calibration graph of XN-C. D3-XN-C concentration was calculated using the above equation.

# Calibration graphs for SIDA

**Figure 16:** Calibration of isoxanthohumol

**Figure 17:** Calibration of Isoxanthohumol-C

**Figure 18**: Calibration of 8-Prenylnaringenin

**Figure 19:** Calibration of 6-Prenylnaringenin

**Figure 20**: Calibration graph of xanthohumol

**Figure 21**:Calibration graph of Xanthohumol-C

# References

Lee, D.-w., and Yun, J. (2005). Direct synthesis of Stryker’s reagent from a Cu(II) salt. *Tetrahedron Lett.* 46(12)**,** 2037-2039. doi: 10.1016/j.tetlet.2005.01.127.

Mahoney, W.S., Brestensky, D.M., and Stryker, J.M. (1988). Selective hydride-mediated conjugate reduction of .alpha.,.beta.-unsaturated carbonyl compounds using [(Ph3P)CuH]6. *J. Am. Chem. Soc.* 110(1)**,** 291-293. doi: 10.1021/ja00209a048.

Roehrer, S., Behr, J., Stork, V., Ramires, M., Medard, G., Frank, O., et al. (2018). Xanthohumol C, a minor bioactive hop compound: Production, purification strategies and antimicrobial test. *J. Chromatogr. B* 1095**,** 39-49. doi: 10.1016/j.jchromb.2018.07.018.

Stevens, J.F., Ivancic, M., Hsu, V.L., and Deinzer, M.L. (1997). Prenylflavonoids from Humulus lupulus. *Phytochemistry* 44(8)**,** 1575-1585. doi: 10.1016/s0031-9422(96)00744-3.

Urmann, C., and Riepl, H. (2020). Semi-Synthetic Approach Leading to 8-Prenylnaringenin and 6-Prenylnaringenin: Optimization of the Microwave-Assisted Demethylation of Xanthohumol Using Design of Experiments. *Molecules* 25(17)**,** 4007. doi: 10.3390/molecules25174007.

Wilhelm, H., and Wessjohann, L.A. (2006). An efficient synthesis of the phytoestrogen 8-prenylnaringenin from xanthohumol by a novel demethylation process. *Tetrahedron* 62(29)**,** 6961-6966. doi: 10.1016/j.tet.2006.04.060.
